# Expression of a Diverse Array of Ca^2+^-Activated K^+^ Channels (SK1/3, IK1, BK) that Functionally Couple to the Mechanosensitive TRPV4 Channel in the Collecting Duct System of Kidney

**DOI:** 10.1371/journal.pone.0155006

**Published:** 2016-05-09

**Authors:** Yue Li, Hongxiang Hu, Michael B. Butterworth, Jin-Bin Tian, Michael X. Zhu, Roger G. O’Neil

**Affiliations:** 1 Department of Integrative Biology and Pharmacology, The University of Texas Health Science Center at Houston, Houston, TX, 77030, United States of America; 2 Department of Cell Biology, University of Pittsburgh School of Medicine, Pittsburgh, PA, 15261, United States of America; Indiana University School of Medicine, UNITED STATES

## Abstract

The voltage- and Ca^2+^-activated, large conductance K^+^ channel (BK, maxi-K) is expressed in the collecting duct system of kidney where it underlies flow- and Ca^2+^-dependent K^+^ excretion. To determine if other Ca^2+^-activated K^+^ channels (KCa) may participate in this process, mouse kidney and the K^+^-secreting mouse cortical collecting duct (CCD) cell line, mCCDcl1, were assessed for TRPV4 and KCa channel expression and cross-talk. qPCR mRNA analysis and immunocytochemical staining demonstrated TRPV4 and KCa expression in mCCDcl1 cells and kidney connecting tubule (CNT) and CCD. Three subfamilies of KCa channels were revealed: the high Ca^2+^-binding affinity small-conductance SK channels, SK1and SK3, the intermediate conductance channel, IK1, and the low Ca^2+^-binding affinity, BK channel (BKα subunit). Apparent expression levels varied in CNT/CCD where analysis of CCD principal cells (PC) and intercalated cells (IC) demonstrated differential staining: SK1:PC<IC, and SK3:PC>IC, IK1:PC>IC, BKα:PC = IC, and TRPV4:PC>IC. Patch clamp analysis and fluorescence Ca^2+^ imaging of mCCDcl1 cells demonstrated potent TRPV4-mediated Ca^2+^ entry and strong functional cross-talk between TRPV4 and KCa channels. TRPV4-mediated Ca^2+^ influx activated each KCa channel, as evidenced by selective inhibition of KCa channels, with each active KCa channel enhancing Ca^2+^ entry (due to membrane hyperpolarization). Transepithelial electrical resistance (TEER) analysis of confluent mCCDcl1 cells grown on permeable supports further demonstrated this cross-talk where TRPV4 activation induce a decrease in TEER which was partially restored upon selective inhibition of each KCa channel. It is concluded that SK1/SK3 and IK1 are highly expressed along with BKα in CNT and CCD and are closely coupled to TRPV4 activation as observed in mCCDcl1 cells. The data support a model in CNT/CCD segments where strong cross talk between TRPV4-mediated Ca^2+^ influx and each KCa channel leads to enhance Ca^2+^ entry which will support activation of the low Ca^2+^-binding affinity BK channel to promote BK-mediated K^+^ secretion.

## Introduction

The kidney is the primary organ for maintaining K^+^ homeostasis of the body. This is accomplished by closely regulating K^+^ excretion to match K^+^ intake under normal physiological states. Renal control of K^+^ secretion occurs predominantly in the late distal tubule, notably the connecting tubule (CNT) and cortical collecting duct (CCD), where K^+^ secretion is tightly controlled [1–6]. This is thought to be mediated by two types of K^+^ channels: the renal outer medullary K^+^ channel (ROMK, Kir1.1), often called the kidney small conductance K^+^ channel [7, 8], and the large- or big-conductance, voltage- and Ca^2+^-activated K^+^ channel (BK, maxi-K^+^ channel; [9–13]). It is general considered that the ROMK channel plays a dominant role in maintaining basal levels of K^+^ secretion. In contrast, the BK channel activity is typically low under basal conditions, but is rapidly stimulated during certain stressed states. This is particularly apparent during states of enhance tubular flow to the distal nephron where BK-mediated K^+^ secretion gives rise to the phenomena of flow-dependent K+ excretion that typically leads to K^+^ wasting and hypokalemia [14–18].

The phenomenon of flow-dependent K^+^ excretion is now known to be a Ca^2+^-dependent process associated with flow-induced Ca^2+^ entry into the distal tubule cells of the collecting duct system (CDS), notably the CNT and CCD [19–22]. Our laboratory [17, 21, 23] and others [19, 22] have shown that elevated flow rates/shear stress activate the mechanosensitive TRPV4 channel in these segments, leading to rapid influx of Ca^2+^ with subsequent activation of BK to give rise to flow-dependent K^+^ secretion. Whether the BK channel is the only Ca^2+^-activated K^+^ channel (KCa) associated with control of K^+^ excretion under these states is currently not known. Indeed, it has been shown in knockout models of the BKα subunit (the channel forming subunit of BK) or some of the associated β subunits [14–16], that flow-induced K^+^ secretion is markedly impaired in these models, typically returning K^+^ excretion rates back towards the basal secretory rates thought to be associated with ROMK. However, it has also recently been shown that elevated distal flow rates lead to enhanced release of ATP into the tubular lumen [24, 25] which, in turn, may impair ROMK activity since luminal ATP is known to inhibit ROMK [26]. Most recently we showed that SK3 is also expressed in the mouse CNT and CCD and, again, was found to be linked to TRPV4 activation including during application of shear stress to cells of split-opened CCD [23] or during application of hypotonic swelling states to CCD M-1 cells [27]. Hence, the participation of SK3 and other KCa channels in regulation of K^+^ secretion in the distal tubule remains largely unknown.

The purpose of the current study was to determine which KCa channels may be expressed in the late distal tubule and play a role in Ca^2+^-dependent processes in the CNT and CCD. Our focus was specifically on those channels that are linked to the TRPV4 channel via TRPV4-mediated Ca^2+^ entry. Our laboratory [17, 21, 28] and others [19] have shown that TRPV4 is the dominant mechanosensitive Ca^2+^-permeable channel expressed in the CNT and CCD and that it underlies Ca^2+^ activation of flow-dependent K^+^ secretion [19, 21, 23, 29]. We demonstrate that, in addition to BK (KCa1.1), an array of KCa channels are expressed in CNT and CCD including SK1 (KCa2.1), SK3 (KCa2.3) and the IK channel (KCa3.1) and, using a model CCD cell line of K^+^ secretion, the mCCDcl1 cell line [30], that these channels are tightly coupled to TRPV4 activation, displaying strong cross-talk among channels. The study opens up a new realm of renal Ca^2+^-activated K^+^ channel diversity and function that may play a critical role in control of membrane potential and/or K^+^ secretion in flow-sensitive segments of the collecting duct system (CDS) of the kidney.

## Materials and Methods

### Animals

C57BL/6 mice were maintained on a normal diet with free access to water. Kidneys were removed and used for experimentation as outlined for each protocol below. All studies were carried out in strict accordance with recommendations in the Guide for the Care and Use of Laboratory Animals of the NIH. All animal protocols were approved by the Institute for Animal Care and Use Committee of The University of Texas Health Science Center (AWA#: A3414-01).

### Cell Culture

mCCDcl1 cells (mouse kidney CCD cell line, [30, 31]) were grown to confluency before use unless otherwise defined in each protocol. In general, cells were grown for 3–5 days on coverslips and 7–9 days on permeable supports at 37°C in “**complete growth medium**” containing DMEM/F12 medium (Life Technology, 21041–025) with addition of the following supplements: 2% fetal calf serum, 0.87 μM insulin, 5 μg/ml human apotransferrin, 10 ng/ml EGF, 1 nM T3, 30 nM dexamethasone and 1% Penicillin/Streptomycin. Before the experiments, the medium for the mCCDcl1 cells on permeable supports was switched to a “**base medium”** (DMEM/F12 with no added supplements) for 24 hrs or longer as outlined below for each experimental protocol.

### Real-Time Quantitative RT-PCR

Total RNAs were prepared from cultured mCCDcl1 cells or whole mouse kidney using TRIzol reagents (Invitrogen) following the manufacturer’s instruction as described previously [23, 32, 33]. All RNA samples were pretreated with DNase I to eliminate potential genomic contamination. The Verso cDNA Synthesis Kit (Thermo Fisher Scientific) was used to synthesize all cDNAs. Quantitative RT-PCR was performed on Eppendorf Mastercycler ep realplex by using KAPA SYBR FAST qPCR Master Mix (Kapa Biosystems). Primer information is provided in Table 1. PCR cycling included initial denaturation at 95°C for 3 min, then 45 cycles of denaturation at 95°C for 8 s each, primer annealing at 60°C for 30 s, and extension of product at 72°C for 30 s, followed by a melting curve for 15 s at 95°C and 15 s at 60°C. Relative gene expression were calculated using comparative C_T_ method (2^-ΔΔCT^ method) and presented as the mRNA level relative to a marker gene mRNA. All studies were performed in triplicate samples.

**Table 1:** Listing of ion channels evaluated by qPCR and the forward and reverse primers employed in the study for each channel.

Gene	Primer Sequence (F: Forward; R: Reverse)
AQP2	F: ATGTGGGAACTCCGGTCCATA
	R: ACGGCAATCTGGAGCACAG
TRPV2	F: TGCTGAGGTGAACAAAGGAAAG
	R: TCAAACCGATTTGGGTCCTGT
TRPV4	F: ATGGCAGATCCTGGTGATGG
	R: GGAACTTCATACGCAGGTTTGG
ROMK	F: GTTTGTCACTCACATATTTGGGC
	R: CCTCGACTGTGCATCTACATTG
SK1	F: GAGAGATCCAGCTGTTCTTGG
	R: GTCCACGTGAAGCGATAGTG
SK2	F: TGCCTCGTCTAGAAGCATTG
	R: TCATGGTACCTTTCACAAGCTC
SK3	F: CATCACGTTCCTTTCCATTG
	R: TCTCTGCTTTGGTGAGTTCG
IK1	F: ATTCCGATCACATTCCTGACC
	R: TGTTGAACTCCAGCTTCCG
BKα	F: TGGTGCCCTCGTAATATACTTCA
	R: CAGCTTATCGTTGGCTGCAAT
BKβ1	F: CCTGGGAGTGGCAATGGTAG
	R: CAAAGGCATGGGTACTGGGG
BKβ2	F: CTGCGCTCCTACATGCAGAG
	R: TGCAGGCAAGGGTACTGAGA
BKβ4	F: ACCAACCCCAAGTGCTCCTAT
	R: GAATGGCTGGGAACCGATCTC

### Western Blotting

Using immunblot methods as previously described [21, 34], two times Laemmli Sample Buffer (Bio-Rad) with 5% fresh β-mercaptoethanol was mixed with 1 part of cell pellet, and then heated for 10 min at 95°C with votexing every 3 min during the heating process. The sample was centrifuged at 12,000 rpm for 2 min. The supernatant was laded to 4–15% SDS-PAGE gradient gel, transferred to PVDF membrane, and blocked with 3% nonfat milk for 1 hour at RT. Membranes were incubated with anti-TRPV4 (Alomone, ACC-034), anti-SK1 (Alomone, APC-039), anti-SK3 (Alomone APC-025), anti-IK1 (ThermoFisher PA5-33875) and anti-BKα (Alomone APC-021) antibody at 1:1000, respectively, at 4°C overnight. After washing, membranes were incubated with goat anti-rabbit secondary antibody (Santa Cruz Biotechnology) 1: 2000 for 1 h. All studies were performed in duplicate or triplicate.

### Cell Immunocytochemistry

mCCDcl1 cells were seeded on to Corning Costar^™^ Transwell^™^ Permeable Supports (0.4 μm pore size) in 24 well plates at 1×10^5^ cells/cm^2^ in complete growth medium. After 7–9 days, cell monolayers were washed in PBS for two times and fixed in 100% methanol (chilled at -20°C) for 5 min at RT, followed by three times wash in ice cold PBS for 5 min each as done before [27, 34]. The cells were then blocked in blocking buffer (PBS, 10% goat serum and 0.05% Triton X-100) for one hour. Whereupon primary antibodies were add to the blocking buffer at 1:100 dilution and incubated overnight at 4°C. The following primary antibodies were used: anti-SK1 (Alomone, APC-039), anti-SK3-ATTO-594 (Alomone, APC-025-AR), anti-IK1 (Alomone, ALM-051), anti-BKα (Alomone, APC-021) and anti-TRPV4 (Alomone, ACC-034). After primary antibody incubation, the cells were washed 3 times in PBS, 5 min each, followed by incubation with secondary antibody (Life Technologies, alexa fluor 488 or 594 labeled goat anti-rabbit) in blocking buffer. The cells were then washed another three times in PBS, 5 min each, and mounted with ProLong Gold Antifade Mountant with DAPI (Life Technologies). Cells were imaged at 100x using a Nikon A1R Confocal Laser Microscope or a Zeiss Axioskop 40 microscope equiped with a AxioCam MRm CCD camera.

### Immunohistochemistry of Mouse Kidney Tissues

Standard immunocytochemistry procedures were used to prepare and immunostain kidney tissue as previously described [21, 23]. Mice were anesthetized with isoflurane inhalation. Kidneys were then removed and fixed by 4% poly paraformaldehyde in PBS buffer for 24 hrs, at 4°C. Whereupon the kidney was incubated with 30% sucrose in PBS buffer for 48–72 hrs or until the kidney sank to the bottom of the tube, at 4°C. Fixed kidneys were then embedded with Tissue-Tek O.C.T. Compound, frozen at -20°C and sectioned (5 microns thick, sagittal and transverse sections) with use of an OTF 5000 cryostat (Bright Instruments).

Prior to staining, tissue sections were allowed to warm to room temperature for 30 min. Subsequently, kidney sections were incubated in acetone for 10 min at RT, followed by 10 min in PBS at RT. The tissue was then circled by PAP pen and blocked in blocking buffer (3% BSA in PBS) for 1 hour. Sections were then incubated with appropriate primary antibodies 1:200 overnight at 4°C. Anti-SK1 (Alomone, APC-039), anti-SK3 (Alomone, APC-025-ATTO-594), anti-IK1 (ThermoFisher Scientific, PA5-33875), anti-BKα (Alomone, APC-107 or APC-151) and anti- TRPV4 (Alomone, ACC-034), were used. Following wash of primary antibodies with PBS for 3 times, 5 min each, tissues were incubated with secondary antibody ((Life Technologies, alexa fluor 488 goat anti rabbit) for 1 hour at RT. The kidney sections were then washed again and incubated with Anti-Aquaporin 2-ATTO-550 (Alomone, AQP-002-AO) for 3 hrs at RT or overnight at 4°C. The tissues were then washed another three times in PBS, 5 min each, and mounted with ProLong Gold Antifade Mountant with DAPI (Life Technologies) and imaged at 20x and 40x (oil) with a Zeiss Axioskop 40 microscope equipped with a AxioCam MRm CCD camera.

Expression of KCa channels in PC and IC cells of mouse CCD were determined from the kidney sections using ImageJ (NIH, version 1.49V) to measure the immuofluorescence intensities for each channel type. Using the immunofluorescence 40X images obtained above, both PC and IC cells were identified in the same tubule as AQP2-positive and AQP2-negative cells, respectively, as done before [17, 21]. Specifically, an ROI was drawn around individual PC and IC cells and the immunofluorescence intensity for each cell measured. No attempt was made in these images to define specific sites of localization within the cells, only whether the expression of the channel was evident in the cells particularly near the cell borders (see RESULTS). Typically 1–3 sections were used from different kidneys (2–3 kidneys) with a total of 5–12 cells analyzed for each CCD identified and the results presented as the total number of PC and IC cells analyzed for each KCa channel. Since AQP2 expression in PC is a well-defined channel in the CCD, all sections were double stained for each KCa channel and AQP2 (see above) and the intensities for each KCa channel in PC and IC normalized to AQP2 intensities of PCs.

### Electrophysiological Assessment of TRPV4 and KCa Channel Cross-Talk

Initial patch clamp assessment of KCa activation utilized whole-cell voltage-clamp recordings in mCCDcl1 cells grown on coverslips. However, with the voltage-clamp methods the TRPV4-mediated Ca^2+^-induced K^+^ currents were found to be highly variable and displayed large oscillation that could not readily be interpreted or isolated by selective KCa channel blockers. Hence, we turned to whole-cell current-clamp methods which we found to display more consistent results, especially when TRPV4 was pre-activated prior to allowing Ca^2+^ influx. With this approach we limited the analysis to monitoring changes in membrane potential, Vm, as an index of TRPV4 and KCa activation using selective KCa blockers to separate out which channels were activated by TRPV4-mediated Ca^2+^ influx. The selective blockers used were: iberiotoxin (IbTX; 200 nM) for BKα [35, 36], TRAM-34 (TRAM-34; 300 nM) for IK1 [37], and Apamin (Apa; 300 nM) for SK1/SK3 [38, 39].

Whole-cell current clamp recordings were made from cultured mCCDcl1 cells on glass coverslips (80–100% confluence) using an EPC10 amplifier (HEKA Elektronik). Patch electrodes with a resistance of 3–5 MΩ were pulled from borosilicate micropipettes (Sutter Instrument) and filled with pipette solution containing (in mM): 130 K-methanesulfonate, 7 KCl, 0.05 EGTA, 10 HEPES, 1 Na_2_-ATP, 3 Mg-ATP, 0.05 Na_2_-GTP, pH 7.3 adjusted with KOH, 300 mOsM. mCCDcl1 cells (spindle- or polygonal-like, with apparent three dimensional structures) were targeted under x60 water objective lens equipped in a differential interference contrast microscopy (Olympus BX51WI with OLY-150IR video camera). Upon obtaining a successful seal, mCCDcl1 cells were first voltage clamped at -40 mV. After switching to current clamp, current injection was set to 0, thus the membrane potential recorded at the beginning of each trace represented the resting membrane potential (REM) of the recorded cell. mCCDcl1 cells displayed REM ranging from -25 mV to -55 mV. Cells with REM more positive than -25 mV were rejected. Electrical signals were filtered at 2.9 kHz and digitized at 10 kHz. All recordings were conducted at room temperature (~23°C).

During recordings, cells were continuously perfused by extracellular solutions (ECS). Cells in the control group were initially exposed to normal ECS containing (in mM): 140 NaCl, 5 KCl, 2 CaCl_2_, 1 MgCl_2_, 10 HEPES, 10 Glucose, pH 7.4 adjusted with NaOH, 310 mOsM. They were then sequentially exposed to ECS without 2 mM CaCl_2_ (nominally Ca^2+^ free ECS) for about 1 min, then 10 nM GSK101 in the nominally Ca^2+^ free ECS (see figure legend for additional details of the solution changes). In the nominally Ca^2+^ free ECS, GSK101 induced TRPV4 activation leads to Na^+^ influx and, in turn, depolarization of Vm. This perfusion was continued until the TRPV4-mediated depolarization of Vm reached a plateau. Whereupon, the perfusate was rapidly switched to normal ECS (with 2 mM Ca^2+^) with the same concentration (10 nM) of GSK101 for 3 min. This caused an instantaneous hyperpolarization of Vm due to activation of KCa channel by Ca^2+^ influx mediated by TRPV4. To determine the contributions of individual groups of KCa channels, KCa channel blockers (200 nM IbTX, 300 nM TRAM-34, or 300 nM apamin) were applied within 2–3 sec following the exposure of the cell to normal ECS with GSK101 when the instantaneous hyperpolarization was fully developed. The cell was continuously exposed to 10 nM GSK 101 and the specific blocker in normal ECS for at least 3 more min to allow Vm to increase (depolarize) due to blockade of the KCa channels.

Given the considerable variations of TRPV4 and KCa channel expression levels in individual mCCDcl1 cells, the instantaneous hyperpolarization of Vm (first hyperpolarization peak) that occurred during the initial 2–3 sec application of normal ECS with GSK101, which represented the overall response of KCa channels to TRPV4-mediated Ca^2+^ influx, was found to be quite variable among recorded cells. Therefore, the Vm value obtained at 3 min following administration of the blockers (indicated by the vertical arrowheads in the data figure) were normalized to the “first hyperpolarization peak” (indicated by the dashed line in data figure) of the same cell for quantification of the effects of individual KCa channel blockers.

Data are presented as mean±SE, where n is the number of cells analyzed. The data were analyzed by one-way ANOVA followed by Dunnett post hoc tests for comparison to the control group. A significant difference was defined as p<0.01 (**).

### Measurement of Intracellular Calcium

Ratiometric fluorescence imaging of fura-2 was used to monitor intracellular Ca^2+^ levels, [Ca^2+^]_i_, in mCCDcl1 cells using standard procedures as done extensively before [17, 27]. Briefly, cultured cells on coverslip chips were loaded with fura-2/AM (2 μM) for 40 min, washed, placed on the coverslip bottom of an open perfusion chamber (0.5 ml), and imaged with an InCa Imaging Work station (Intracellular Imaging, Inc.) at 37°C using a 20× Nikon Super Fluor objective. [Ca^2+^]_i_ was estimated from the fura-2 fluorescence by excitation at 340 nm and 380 nm and calculating the ratio of the emission intensities at 511 nm in the usual manner every 5 s. Results are subjected to intracellular fura-2 calibration and the ratios converted to [Ca^2+^]_i_ activity as described by Grynkiewicz et al. [40] using methods outlined previously [17, 27]. Typically, from 8–10 cells were assessed for [Ca^2+^]_i_, on each coverslip with 3–4 coverslips used per treatment group. The data are typically presented as total number of cells analyzed for each treatment group.

### TEER Measurement

Transepithelial electrical resistance (TEER) measurements were made using an EVOM meter (World Precision Instruments) and chopstick-style electrodes as done previously [41] and by others [42]. Briefly, mCCDcl1 cells were seeded on to Corning Costar^™^ Transwell^™^ Permeable Supports (0.4 μm pore size) in 24 well plates at 1×10^5^ cells/cm^2^ in complete growth medium. After 7–9 days, cell monolayer formation in transwell cups was assessed by measuring TEER where all monolayers reached TEER values of 1000 Ω cm^2^ or more, which was taken as an index of achieving monolayer confluency. Whereupon, the cells were incubated in base medium for another 2 days before performing the experiments. All TEER measurements were made at 37°C in base media. Under these conditions the monolayers displayed an average TEER reading of 3081 ± 155 Ω cm^2^ (n = 15) during basal conditions.

### Chemicals

The following chemicals were used in this study: GSK101 (GSK1016790A, Santa Cruz Biotechnology) from a stock solution (1 mM) in DMSO; apamin (Apa, Alomone) from a stock solution (1 mM) in PBS; iberiotoxin (IbTX, Alomone) from a stock solution (0.1 mM) in PBS; TRAM 34 (TOCRIS) from a stock solution (10 mM) in DMSO; and Tertiapin-Q (TQ, Alomone) from a stock solution (10 μM) in PBS.

### Statistical Methods

Summary data are presented as mean values ± SEM. “n” is either the number of cells analyzed or the number of sections analyzed as described in the figure legends. Where appropriate, differences among groups of studies were analyzed by either a *t*-test or a one-way ANOVA, followed by the *a posteriori* Dunnett’s test, as appropriate. For all analysis the significance level was set at P<0.05 unless otherwise indicated in the figure legends.

## Results

### K^+^ Channel Expression in mCCDcl1 Cells and Mouse Kidney

**Fig 1:**
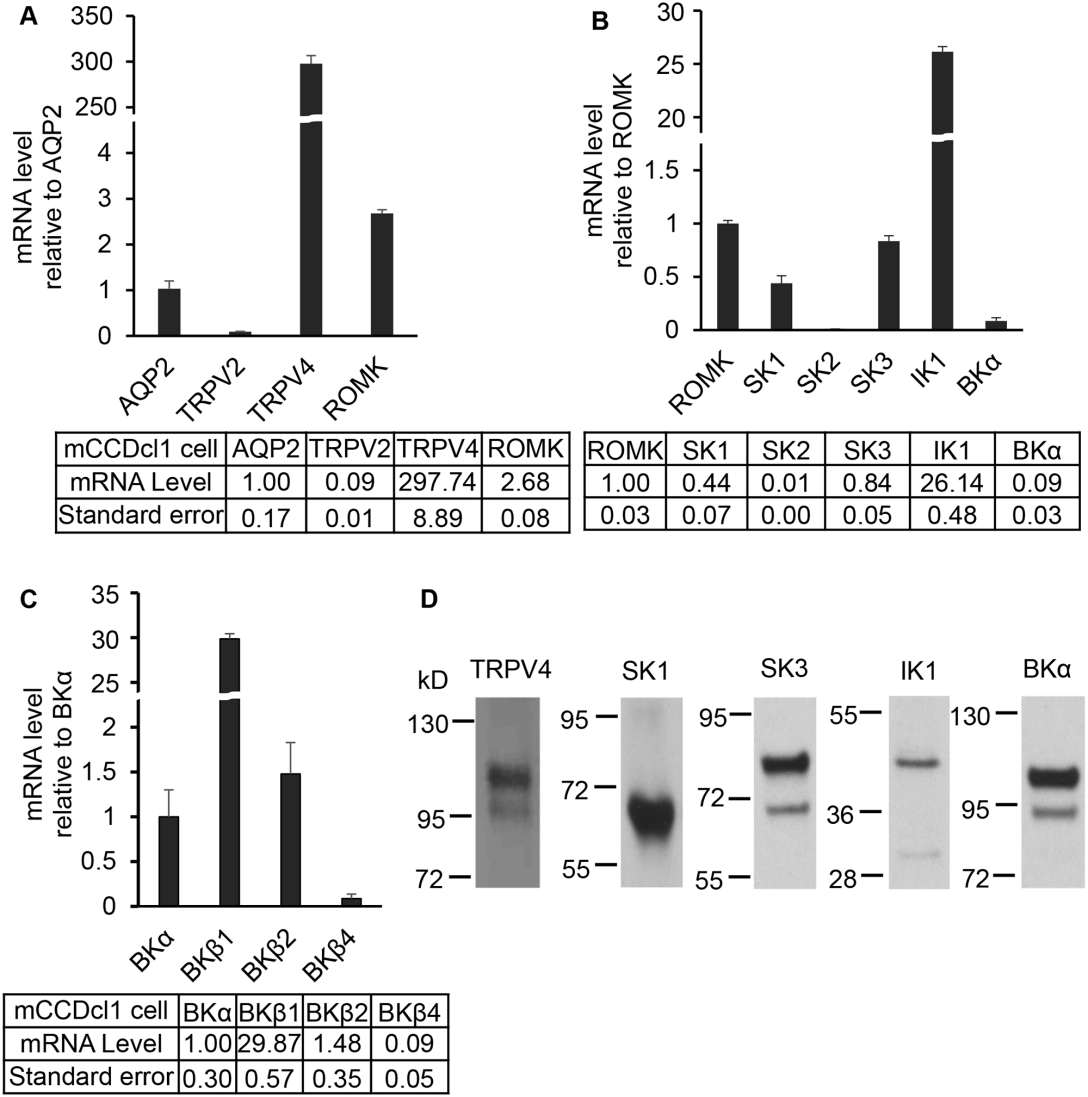
Differential mRNA and protein expression levels of ion channels in mCCDcl1 cells. **A.** qPCR analysis of mRNA expression levels of marker channels relative to the AQP2 water channel. The mechanosensitive TRPV4 channel mRNA levels and ROMK mRNA levels are relatively high while a second mechanosensitive TRP channel, TRPV2, are very low. For comparison, mRNA expression levels for all channels are also given, relative to AQP2 mRNA levels, in the accompanying Table 1. **B.** qPCR mRNA expression levels of KCa channels relative to the ROMK channel. It shows that SK1, SK3, IK1 are expressed at relative high mRNA levels, BKα at moderate mRNA levels, and SK2 at low mRNA levels. **C.** mRNA expression of BK channel subunits relative to BKα mRNA levels. It shows high relative mRNA levels for BKβ1 and BKβ2, but relatively low levels for BKβ4. **D.** Immunoblots of mCCDcl1 cells for the key channels showing appropriate protein bands for TRPV4 (98 kD), SK1 (64 kD), SK3 (81 kD), IK1 (45 kD), and BKα (110 kD).

In initial studies we tested for the expression of KCa channels, including SKs, IK, and BK channels, as one or all of these channels could potentially underlie Ca^2+^-activated processes in the collecting duct system. Further, since we have shown that TRPV4 is a key mechanosensitive Ca^2+^ entry TRP channel in the distal tubule [17, 21, 23], its’ expression was also assessed. Initial studies were performed in the mCCDcl1 cell line as it has been shown that these cells are a potential good model for CCD K^+^ secretion [30] whereas other CCD cell lines, e.g. M-1 cells [43] and mpkCCD cells [30], do not appear to support transepithelial K^+^ secretion. Cells were grown to confluency in culture dishes and RNA harvested and analyzed by quantitative PCR (qPCR) as summarized in Fig 1. Using aquaporin 2 (AQP2) as our initial reference channel for the CDS, it is apparent from the data in Fig 1A that TRPV4 and ROMK are both highly expressed in mCCDcl1 cells, averaging 297 and 2.7 fold higher mRNA levels than that observed for AQP2 expression. Expression of a second mechanosensitive TRP channel, TRPV2, which we have shown to be expressed in CCD M-1 cells [27], was found to be expressed at very low levels in mCCDcl1 cells.

Comparison of expression of KCa channels in mCCDcl1 cells demonstrated significant expression of an array of KCa channels. As shown in Fig 1B, comparing expression of the KCa channels to ROMK, a distal tubule marker of K^+^ channels, revealed relatively high mRNA levels for SK1 (0.44 fold), SK3 (0.84 fold) and IK1 (26.1 fold), but not for SK2 (0.01 fold). While BKα expression appeared to be modest (0.09 fold relative to ROMK), this is a large channel that can support large K^+^ currents with only a few channels being expressed [11].

Finally, we also evaluated expression of the BKα accessory β subunits in mCCDcl1 cells since the subunits can modulate BKα activation. As shown in Fig 1C, BKβ1 and BKβ2 were highly expressed, relative to BKα, with mRNA levels averaging 29.9 and 1.5 fold higher than that for BKα, respectively. In contrast, BKβ4 displayed more limited expression levels (0.09 fold).

The key channels discovered by qPCR were also assessed for expression of channel proteins using immunoblots of mCCDcl1 cell lysates. As shown in Fig 1D, immunoblot analysis revealed protein bands at the expected levels for TRPV4, SK1, SK3, IK1 and BKα. Hence, these specific channels appear to be expressed at both the mRNA and protein levels and, hence, are likely to be functional channels in mCCDcl1 cells (see below).

**Fig 2:**
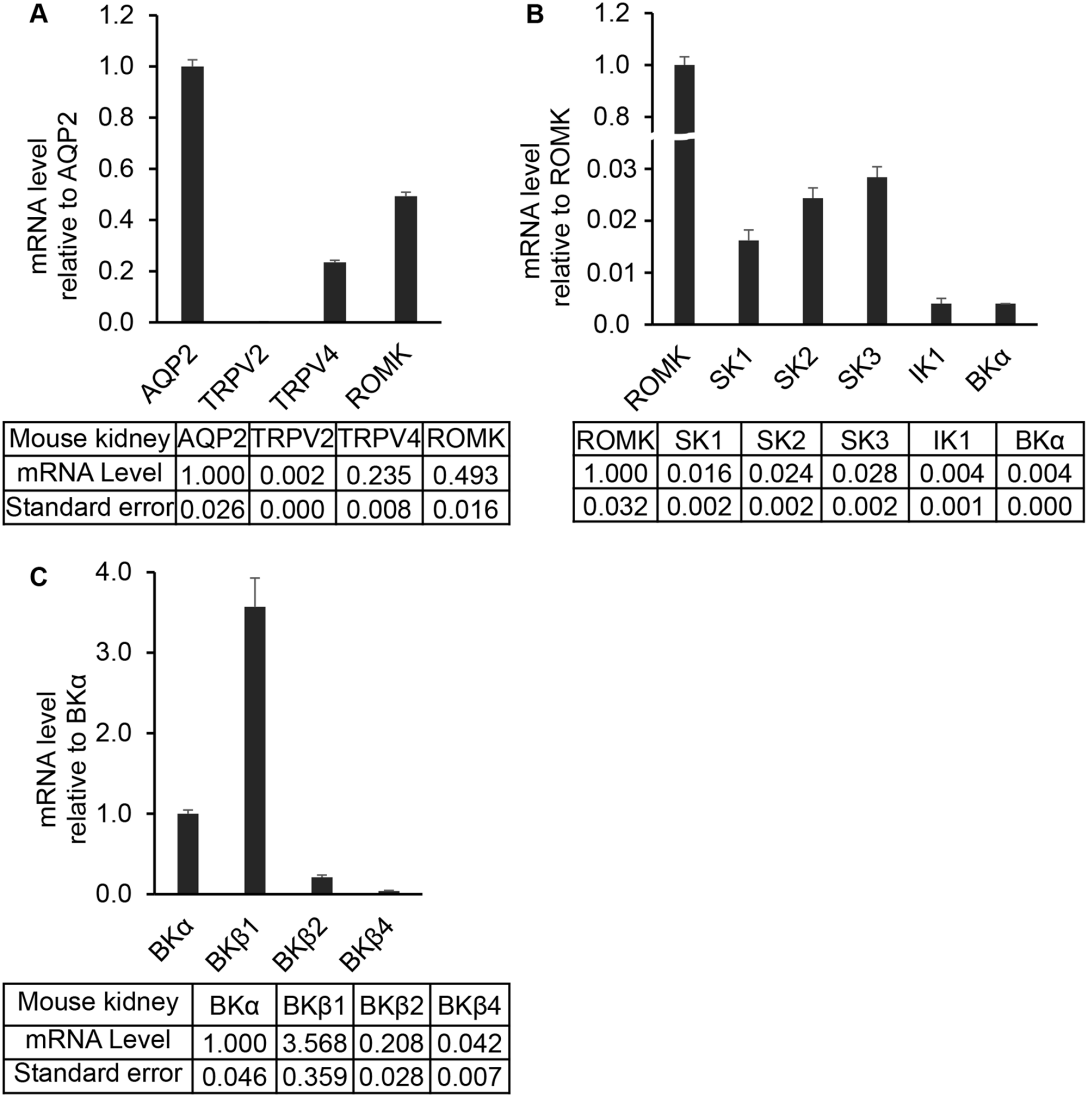
Differential mRNA expression levels of ion channels in mouse kidney. **A.** qPCR anlysis of mRNA expression levels of marker channels relative to the AQP2 water channel. As in mCCDcl1 cells, the mechanosensitive TRPV4 channel mRNA levels and ROMK mRNA levels are relatively high while mRNA levels for a second mechanosensitve TRP channel, TRPV2, are very low. For comparison mRNA expression levels for all channels are also given, relative to AQP2 mRNA levels, in the accompanying Table 1. **B.** qPCR analysis of mRNA expression levels of KCa channels in mouse kidney relative to the ROMK channel. It shows that SK1, SK2, and SK3 are expressed at relative high mRNA levels while IK1 and BKα are expressed at moderate mRNA levels. **C.** mRNA expression of BK channel subunits relative to BKα mRNA levels. The results show high relative mRNA levels for BKβ4, moderate mRNA levels for BKβ2, and low mRNA levels for BKβ4.

To evaluate whether the channels identified in mCCDcl1 cells may be expressed in the kidney, we extended the qPCR analysis to RNA isolated from the mouse kidney (mkidney). Relative to AQP2 mRNA expression, both the TRPV4 and ROMK mRNA were expressed in kidney, averaging 0.23 and 0.49 fold relative to AQP2 mRNA levels (Fig 2A). Again, TRPV2 was observed to be poorly expressed. Upon comparison of the kidney KCa channel mRNA expression levels, relative to ROMK (Fig 2B), all three small conductance channels were observed to be expressed at moderately low levels (SK1: 0.02 fold, SK2: 0.02 fold, and SK3: 0.03 fold), as was IK1 (0.004 fold) and BKα (0.004 fold). While expression at the kidney mRNA level for the KCa channels may appear low relative to ROMK, it is recognized that using whole-kidney RNA provides a very high background for detecting these channels which, if expressed only in the CNT and CCD, would be expected to display relatively low mRNA levels relative to other channels, including ROMK since ROMK it is also expressed in other tubular segments. Indeed, functional BKα channels have been well established as a key channel in the CNT and CCD [9, 10, 14–16] with our more recent studies pointing to a potential role for SK3 function in the CNT and CCD [23]. Hence, while expression levels of these channels may appear to be low relative to ROMK mRNA levels, functional channels can still be expressed in specific nephron segments. Finally, analysis of BKβ subunit expression, relative to BKα, demonstrates relatively high levels of expression of BKβ1 (4.5 fold) and BKβ2 (0.5 fold) similar to that noted for mCCDcl1 cells. In contrast, BKβ4 expression was found to be moderate (0.001 fold), although it is known to be a functional subunit in a subset of CCD cells, the intercalated cells [44, 45]. The results from the mouse kidney are similar to that reported by others [46].

### Immunofluorescence Localization of TRPV4 and KCa Channels

**Fig 3:**
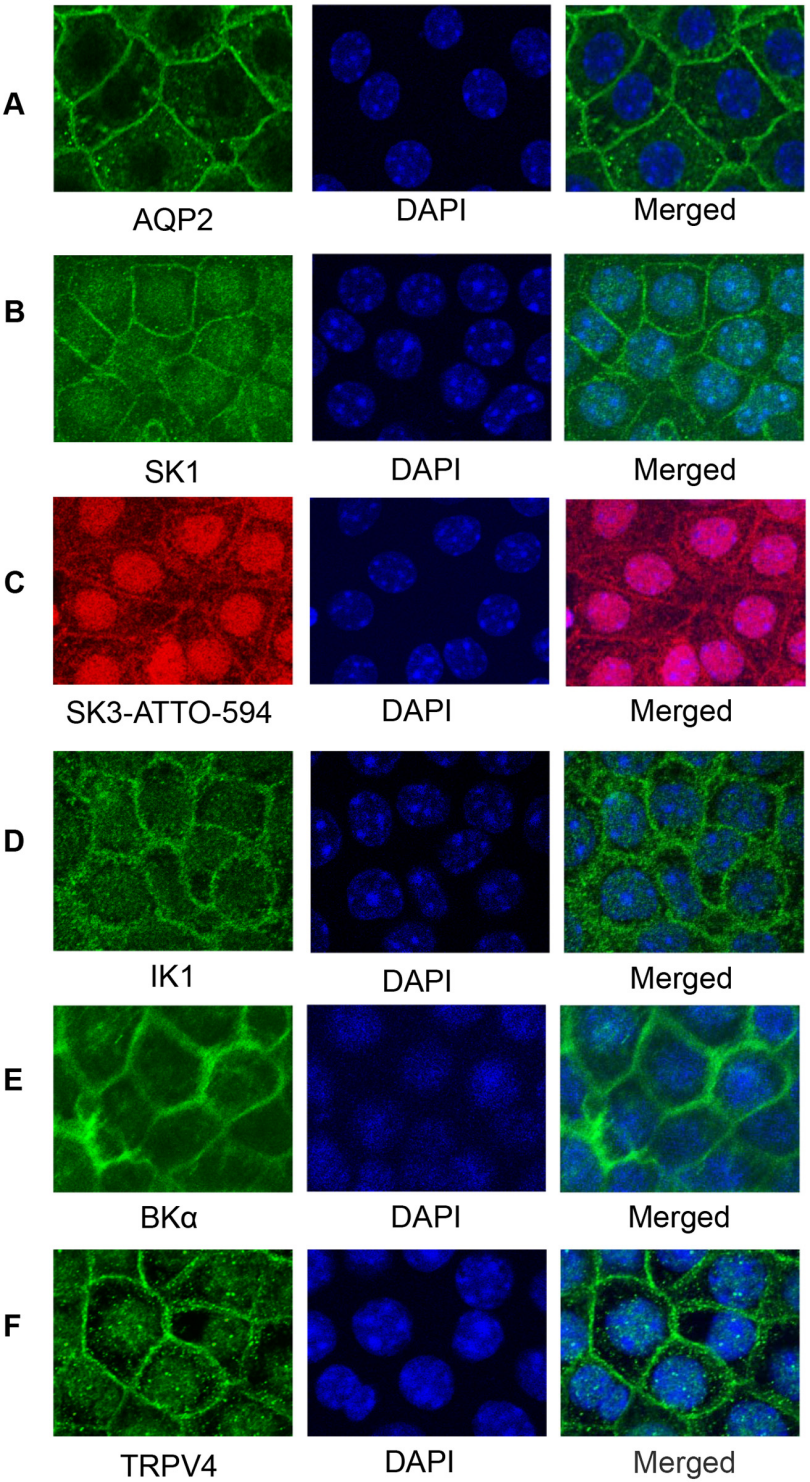
Confocal immunocytochemistry fluorescence images showing results of staining for ion channels in mCCDcl1 cells grown on Transwell^™^ Permeable Supports. Results show immunostaining for the ion channel of interest and for nuclei (DAPI). **A.** AQP2 staining of cells (green, 488 nm) shows prominent staining along the plasma membrane with low staining within the cytosol. **B.** SK1 staining of cells (green, 488 nm) shows clear staining along the plasma membrane borders and modest staining within the cytosol. **C.** SK3 staining of cells (red, 594 nm) shows significant staining along the cell borders and within the cytosol. Modest staining of the nuclei is apparent (SK3, Merged). **D.** IK1 staining (green, 488 nm) is modest along the plasma membrane borders and within the cytosol. **E.** BKα staining of cells (green, 488 nm) with prominent staining of the cell borders and low staining of the cytosol and nuclei. **F.** TRPV4 staining of cells (green, 488 nm) with prominent staining of the cell plasma membrane and modest staining of the cytosol and nuclei. All fluorescence images were obtained at 100x (oil) with a Nikon A1R Confocal Laser Microscope.

The expression profiles for TRPV4 and KCa channels were further assessed by immunocytochemical staining of mCCDcl1 cells. mCCDcl1 cells were grown to confluency on permeable supports or coverslips and immunostained as done before [21, 27]. As shown in the confocal immunofluorescence images in Fig 3A for cells grown on permeable supports, AQP2 showed strong staining along the cell borders. Staining is particularly apparent at sites of cell-cell contact which, as we have shown before [27], is a good index of membrane staining. SK1 likewise displayed prominent staining along the cell border although modest staining was apparent within the cytosol (Fig 3B) which may reflect cytoplasmic pools of SK1. SK3 likewise displayed staining along the cell borders, although more modest, with some staining within the cytosol and the nucleus (Fig 3C). These results point to a likely functional role of both SK1 and SK3 with the noted localization along the plasma membrane (see below). SK2 levels were not evaluated since they were shown to display very low levels of mRNA relative to other SK channels in mCCDcl1 cells (Fig 1).

For the intermediate conductance and large conductance channels, IK1 displayed staining along the plasma membrane with modest staining of the cytosol (Fig 3D). The most prominent staining for KCa channels was observed for BKα localized at, or near, the cell membrane with little staining within the cytosol (Fig 3E). This staining pattern would be consistent with the known localization of BKα in the kidney CNT and CCD, as shown by others ([47–49]) and now by our laboratory (see below). Finally, since TRPV4 is the noted Ca^2+^ permeable channel driving certain Ca^2+^-dependent events in the CNT and CCD, we also evaluated its localization in the mCCDcl1 cells. As shown in Fig 3F, strong TRPV4 staining was apparent at, or near, the plasma membrane with some modest staining within the cytosol. In separate studies we have also found a very similar pattern of staining for TRPV4 and the KCa channels in mCCDcl1 cells grown on glass coverslips (data not shown). Hence, a functional role for TRPV4 in Ca^2+^-dependent signaling and KCa channel activation is likely for mCCDcl1 cells grown on either coverslips or permeable supports.

**Fig 4:**
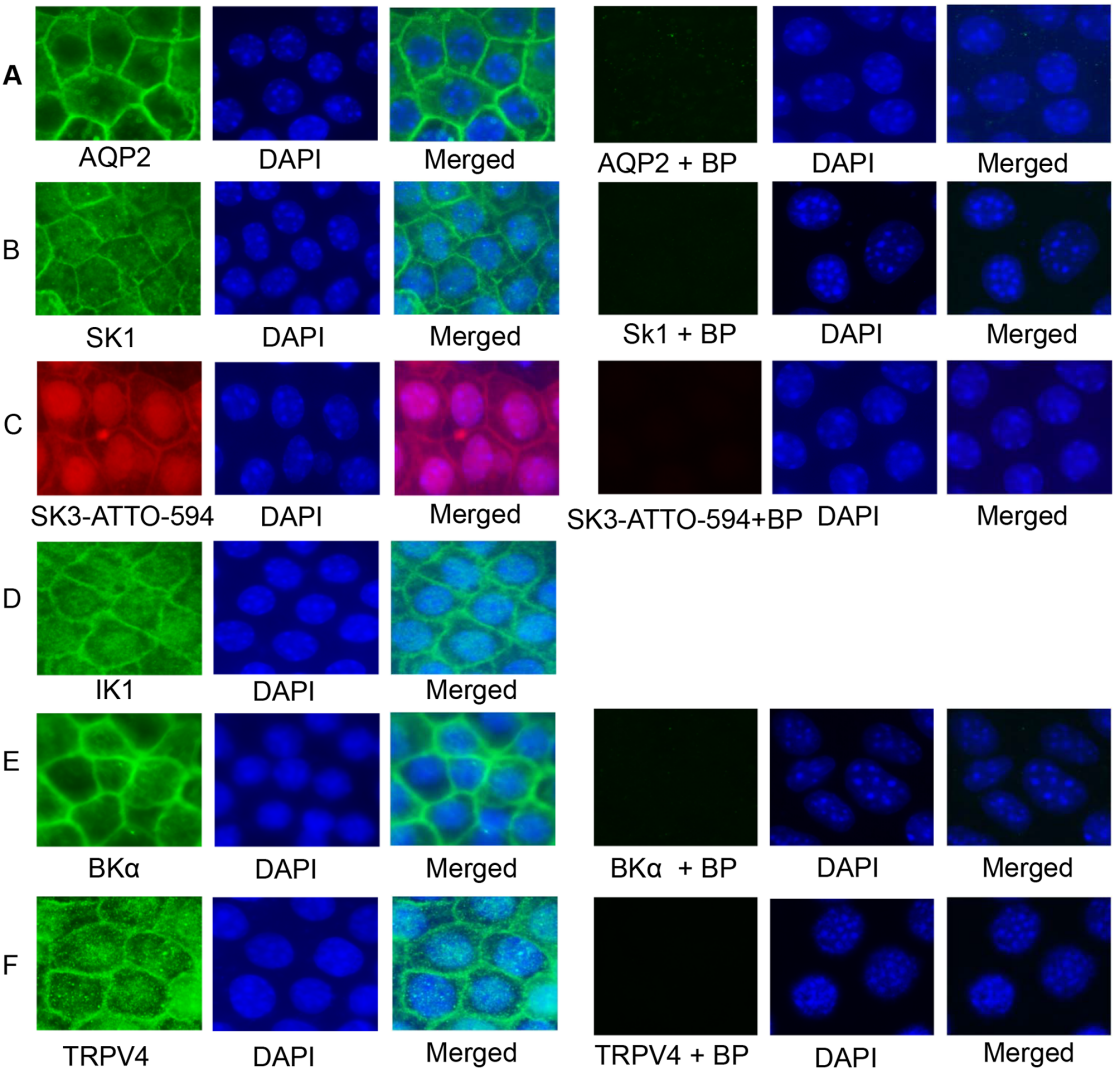
Immunocytochemistry fluorescence images (wide-field fluorescence) of staining for ion channels with or without the control blocking peptides (BP) for the respective primary antibodies. mCCDcl1 cells were grown to confluency on Transwell^™^ Permeable Supports. **A-F.** Left three columns: staining of ion channels without blocking peptides and DAPI staining of the nuclei showed similar localization immunostaining patterns as demonstrated in the confocal images of Fig 3. Right three columns: respective blocking peptides (**+BP**) of the ion channel primary antibodies were used as a control to verify antibody specificity. In each case, incubation with the respective blocking peptide largely abolished the immunostaining thereby demonstrating primary antibody specificity for the channel epitope. The anti-IK1 antibody in **D** is a monoclonal antibody (Alomone ALM05), thereby already providing some specificity, to which a control blocking peptide is not available. All fluorescence images were obtained at 100X (oil) with a Zeiss Axioskop 40 wide-field fluorescence microscope using an AxioCam MRm CCD camera.

To further evaluate the antibody specificity of the antibodies used in this study, additional immunocytochemical staining studies were performed utilizing blocking peptides specific for each primary antibody employed (the anti-IK1 antibody is a monoclonal antibody, ALM05 from Alomone, with no blocking peptide available). The effects of each competing blocking peptide (BP) was performed and monitored by using a Zeiss Axioskop 40 microscope. As shown in Fig 4A–4F, the left three columns demonstrated typically antibody labeling in the absence of BP (normal binding) where localization was found to be similar, as expected, to that observed with the confocal imaging (compare to Fig 3). In contrast, in the presence of competing BP (right three columns), specific channel antibody staining was largely abolished (see column 4, Ab +BP) for each of the channels, thereby providing evidence of specificity of antibodies used for channel localization in these studies.

**Fig 5:**
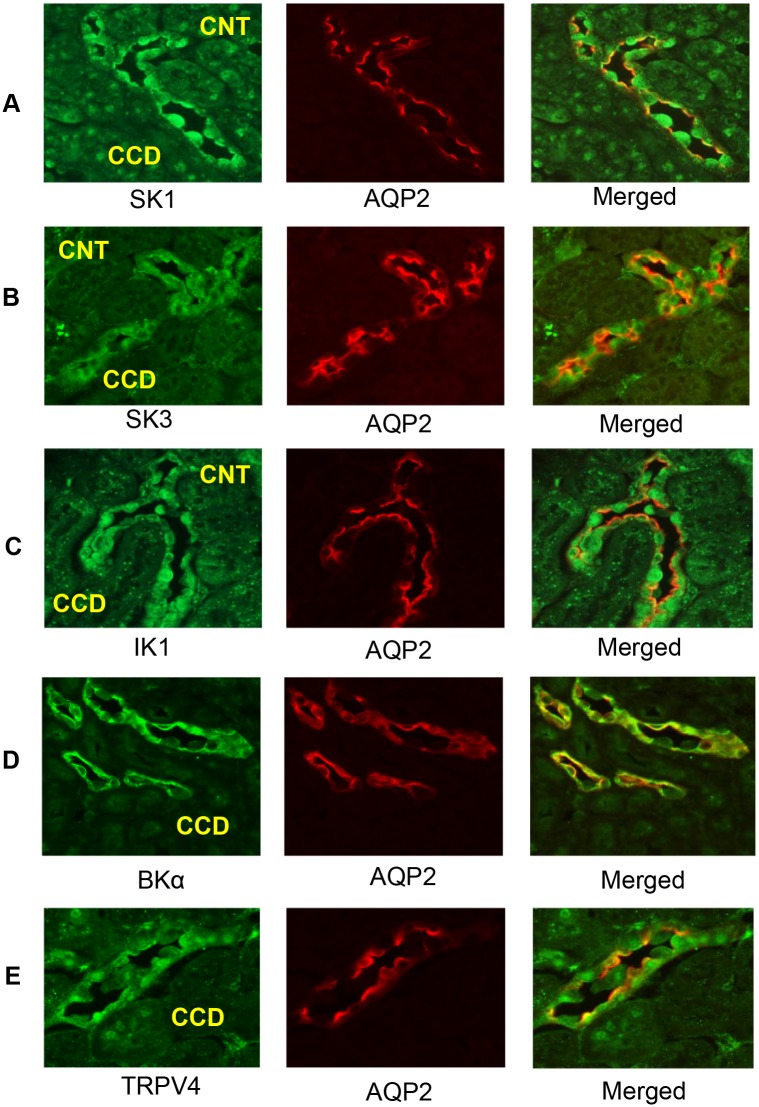
Immunocytochemistry fluorescence images showing results of staining for ion channels in mouse kidney cortical sections at low magnification. Results show staining for the ion channel of interest and for AQP2 (Red, 594 nM), a marker of CNT and CCD in the cortex. A bifurcation of AQP2-positive tubules was used to identify CNT segments (above the bifurcation) and CCD segments (single tubule below the bifurcation). **A.** SK1 staining of tubule segments (green, 488 nm) shows strong staining of many cells along both the CNT and CCD (SK1, labels CNT and CCD). Merged images with AQP2 staining verify the CNT and CCD sites of localization. **B.** SK3 staining of tubule segments (green, 488 nm) shows prominent staining of most cells along both the CNT and CCD (SK3, labels CNT and CCD). Merged images with AQP2 verify the CNT and CCD sites of localization. **C.** IK1 staining (red, 594 nm) is strong in most cells of both the CNT and CCD (IK1, labels CNT and CCD) as verified in the Merged image. **D.** BKα staining (green, 488 nm) is prominent in most cells of both the CCD (BKα, CCD) and along the CNT (data not shown) as verified in the Merged images. **E.** TRPV4 staining (green, 488 nm) is apparent in most cells of the CCD (TRPV4, CCD) and in the CNT (data not shown) as verified in the Merged images. All fluorescence images were obtained at 40x (oil) with a Zeiss Axioskop 40 microscope using a AxioCam MRm CCD camera.

In a similar manner as done for the mCCDcl1 cells, we used immunofluorescence labelling to assess for localization of the ion channels of interest in CNT and CCD of mouse kidney cortical slices. Using AQP2 and TRPV4 staining as markers of CNT and CCD in the cortex, as previously described [17, 23], we demonstrated expression of TRPV4, as before, and the array of KCa channels along both the CNT and CCD. In these studies identification of a CCD bifurcation into two tubules was taken as the division between the upstream CNT and a single downstream CCD. As evident, both SK1 (Fig 5A) and SK3 (Fig 5B) stained strongly along the length of both the CNT and the CCD (note tubule labels for CNT and CCD). Positive co-staining for AQP2 confirmed the localization to the CNT and CCD. The localization of SK3 to these segments also confirms our earlier analysis of SK3 expression in these segments of the mouse CDS [23]. In a similar manner, localization of IK1 (Fig 5C) and BKα (Fig 5D) staining to the AQP2-positive segments verifies the expression of these Ca^2+^-activated K^+^ channels along the CNT and CCD. TRPV4 expression, as anticipated, was also found to be highly expressed along the length of both the CNT and CCD (Fig 5E), as shown previously [17, 21].

**Fig 6:**
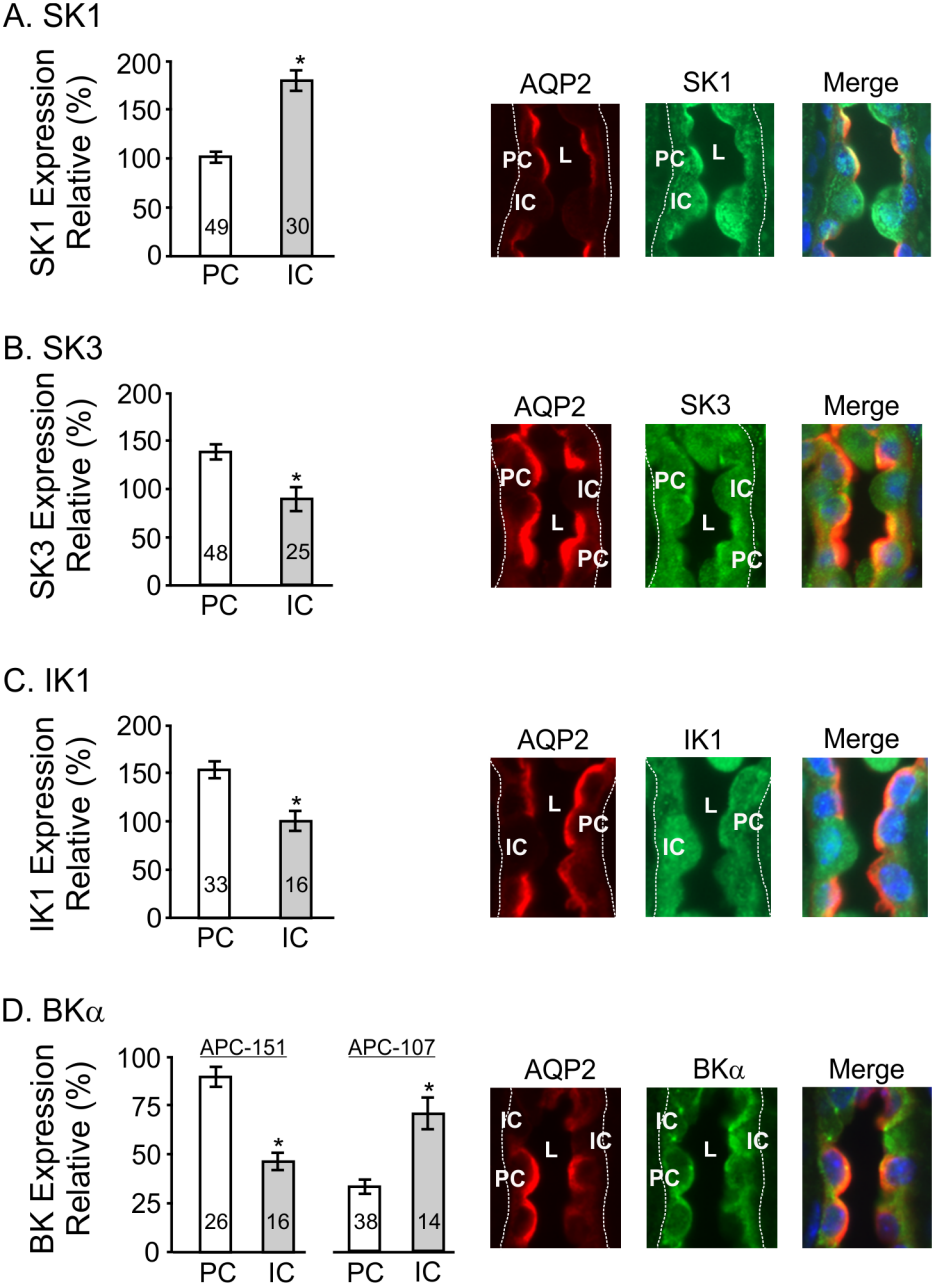
Differential expression of ion channels between principal cells (PC) and intercalated cells (IC) in the mouse kidney CCD. AQP2 staining was used to identify PC, the AQP2-positive cells, and IC, the APQ2-negative cells. Bar graphs give the mean ± SEM for the normalized intensities for each KCa channel for both PC and IC. The number of PC and IC cells analyzed, n, is indicated on the bar graphs. The images of CCD show representative immunofluorescence examples for each KCa channel and AQP2 (40X) where one or two PC (AQP2-positive) and IC (AQP2-negative) cells are labelled as “PC” or “IC.” The outer border of the tubule in each image is indicated by the dashed white line (indicating the basal side or anti-luminal side of tubular cells). “L” identifies the tubular lumen. The immunofluorescence intensity was determined for each KCa channel using ImageJ and normalized to the intensity levels of AQP2 expression in PC (see Methods). Each example gives staining for AQP2 (red), the KCa channel (green), and the merged image (AQP2, KCa channel) that includes DAPI staining to identify nuclei. **A.** Expression of SK1 in PC and IC showing dominant relative expression in IC over PC (P<0.001). SK1 expression was apparent within the cytosol and along the luminal border of both PC and IC as apparent in the representative images. Light staining along the basolateral border is also apparent in some cells. **B.** Expression of SK3 in PC and IC showing relatively more dominant expression in PC over IC (P<0.001). SK3 expression was apparent within the cytosol with light expression along the basal aspect of some cells and more dominant expression along the luminal border of PC. **C.** Expression of IK1 in PC and IC showing relatively more dominant expression in PC over IC (P<0.001). IK1 expression was apparent within the cytosol, especially of IC, but also apparent along the luminal border of PC with light staining along the basal aspect of the cells. **D.** Expression of BKα in PC and IC showing strong staining along the luminal border of both PC and IC. However, the intensity of staining between PC and IC was found to vary depending on the primary antibody used for the immunostaining. In our hands, the Alomone APC-151 BKα antibody staining was most apparent in PC over IC (P<0.001, compare bar graph labeled APC-151 with APC-107). In contrast, the Alomone APC-107 BKα antibody staining was most apparent in IC over PC. Since the antibodies were made to different epitopes of the BKα channel, such variations can be anticipated (see text).

Finally, while not the primary focus of the current study, the differential staining of principal cells (PC) and intercalated cells (IC) in the CCD was used to define differential expression of the channels within cell types of the CCD using fluorescence co-localization imaging as done before [17, 23, 27]. Whole cell imaging was evaluated with no attempt to provide an in depth compartmentalization of channels in this initial survey of KCa channel expression in CCD. AQP2 staining was used to identify PC (AQP2-positive) and IC (AQP2-negative) where immunofluorescent intensities were quantified with ImageJ. The results are summarized in Fig 6 for each of the KCa channels. For the immunofluorescent images the outer aspect of the tubule is outlined with dotted white lines and the tubular lumen labeled as “L.” It was found that the KCa channels immunostained both PC and IC, but with apparent differential expression between cell types. SK1 displayed high expression in IC over PC, IC>PC (Fig 6A), while SK3 and IK1 displayed more prominent staining in PC over IC, PC>IC (Fig 6B and 6C, respectively). For each cell type, staining for these channels was apparent within the cytosol and near cell borders of most cells. SK1 expression pattern was more dominant along the luminal border, with minimal apparent staining along the basolateral aspects of both PC and IC. SK3 and IK1, however, showed staining along the luminal border with more modest staining along the basolateral aspect of the cell, confirming our prior results with SK3 [23].

In contrast to the findings with SK1/SK3 and IK1, BKα staining was dominant along the luminal aspect of both PC and IC, but was found to vary between cell types depending on which of two primary antibodies was employed (Fig 6D). Modest staining of the cytosol was typically apparent with little staining along the basolateral aspects of the cells. However, using Alomone’s anti-BKα antibody (Cat #APC-151) for immunolocalization displayed greater staining of PC over IC cells (PC>IC), whereas a second Alomone anti-BKα antibody (Cat #APC-107) showed greater staining in IC versus PC cells (IC>PC, Fig 6D). Differential staining patterns between primary antibodies is not surprising since the two antibodies were made to very different epitopes (one extracellular, one intracellular), although any difference in the epitopes or the antigen binding sites on the antibodies (paratopes), or BKα assembly in PC versus IC, can participate in such differential staining. It should be noted that others have reported much stronger immunostaining of BKα in IC, with little or moderate staining of PC, using other anti-BKα antibodies [47–49] (see Discussion). Finally, we have also assessed TRPV4 expression in PC versus IC where we confirm our earlier studies [17, 23] showing expression in both cell types, but with dominant staining of PC (PC>IC, data not shown).

### Electrophysiological Analysis of KCa Currents Activated by TRPV4-Mediated Ca^2+^ influx

**Fig 7:**
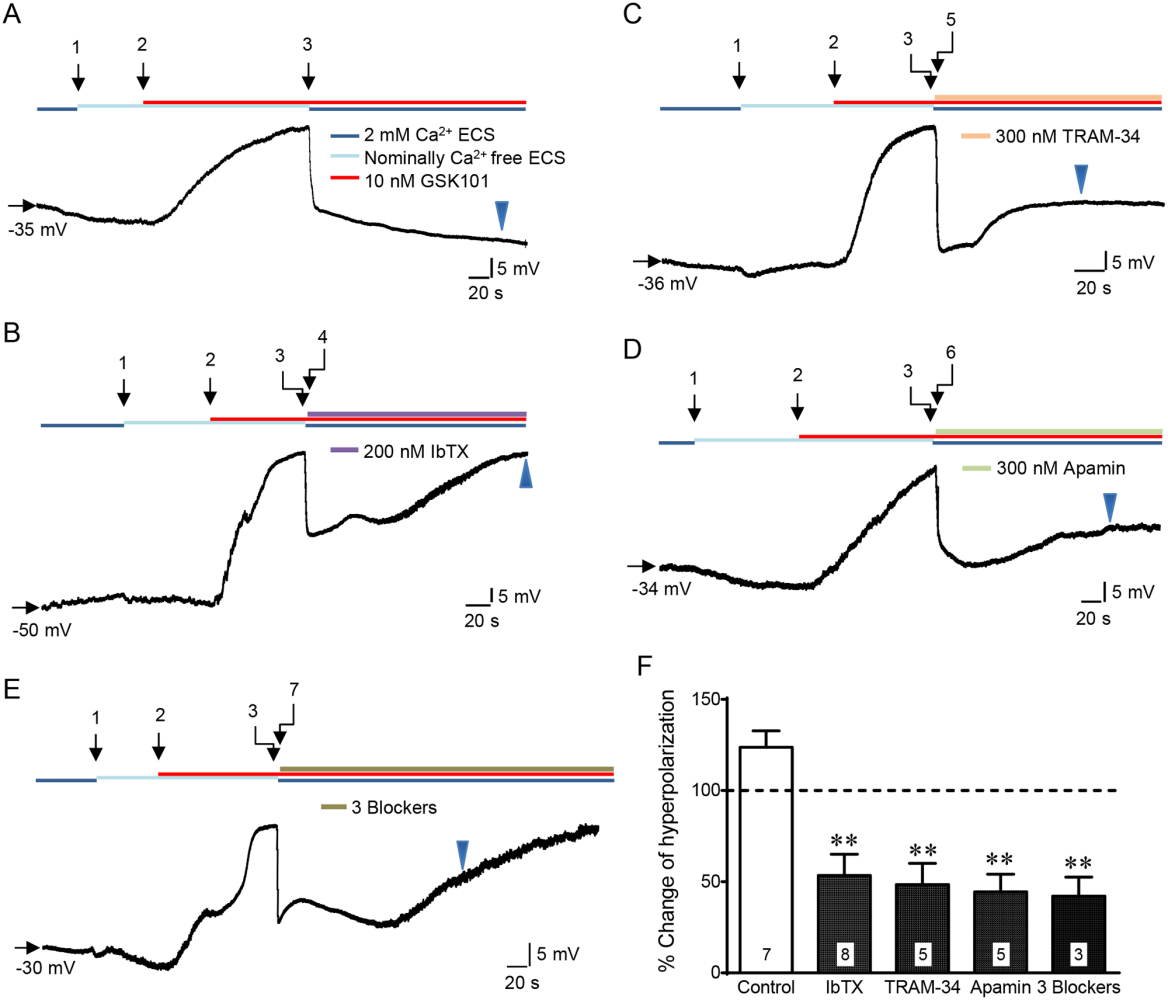
Patch clamp analysis showing membrane hyperpolarization following TRPV4-mediated Ca2+ influx and the effect of KCa channel blockers in mCCDcl1 cells grown on glass coverslips. Current clamp recordings were initiated in ECS with 2 mM CaCl_2_ and 140 nM NaCl (normal ECS) with no current injection. The membrane potential (Vm) recorded at the beginning of each cell measurement is marked by a horizontal arrow. After a stable Vm was achieved, the normal ECS was quickly changed to the ECS without 2 mM Ca^2+^ (nominally Ca^2+^ free ECS, arrow 1), but still with 140 mM NaCl. A slow drift of the Vm (<10 mV) occurred, initially, but stabilized within 1 min. Whereupon, 10 nM GSK101 dissolved in a nominally Ca^2+^-free ECS was applied to the cell (arrow 2) which led to a depolarization of Vm, due to TRPV4-mediated Na^+^ influx into the cell. Once the depolarization reached a new plateau (between -10 mV to 5 mV), the perfusion line was switched to the one with 10 nM GSK101 dissolved in normal ECS (with Ca^2+^, arrow 3), which induced rapid Ca^2+^ influx, and was continued for at least 3 min. The “instant” and significant Ca^2+^ increase in the cytoplasm immediately activated KCa channels which elicited an instant and strong hyperpolarization (20–30 mV) within 2–3 sec (first hyperpolarization peak). This was followed by a slow further hyperpolarization of Vm in normal ECS. If KCa blockers were applied immediately after the first hyperpolarization peak, the blockers lead to a marked reduction in the extent of the Vm hyperpolarization as shown for all KCa blockers (**B-E**). **A.** Representative Vm tracing in a control cell showing that rapid induction of Ca^2+^ influx (arrow 3) lead to a rapid hyperpolarization of Vm within 2–3 sec (first hyperpolarization peak) followed by a second slower hyperpolarization over the remaining 3 min. **B-E.** Representative tracing showing the effect of addition of KCa channel blockers (arrows 4–7) after the first hyperpolarization peak which lead to marked reduction of hyperpolarization response over the subsequent 3-min perfusion period following addition of IbTX (**B**), TRAM-34 (**C**), apamin (**D**), or all three blockers (**E**). **F.** Summary of the blocking effects of KCa blockers where the decrease in the hyperpolarization of Vm was normalized to first hyperpolarization peak response for each cell showing a highly significant reduction in the hyperpolarization response, averaging 53.4 ± 11% for IbTX, 48.4 ± 11.7% for TRAM-34, 44.4 ± 9.7% for Apamin, and 42.1 ± 10.4% for the 3-blockers, **P<0.01 (n’s are given in the bar graph for each blocker).

To determine if the TRPV4 and the KCa channels were functional in the mCCDcl1 cells, we assessed the effects of activating TRPV4 on membrane potential (Vm) changes by whole-cell current-clamp analysis. Using cultured cells grown to confluency on glass coverslips, successful patches could often be maintained for several minutes, allowing for testing the effects of the highly selective TRPV4 agonist, GSK1016790A (GSK101, [27, 50]), and the subsequent Ca^2+^ influx on Vm changes. Further, separate immunostaining of mCCDcl1 cells grown on coverslips showed a very similar pattern of expression for TRPV4 and KCa expression as that observed for cell grown on permeable supports (data not shown; see Fig 3), demonstrating potential functional channels in these cells grown on either support. Under whole-cell current clamp model without current injection, the resting membrane potential was determined to average -32.7 ± 1.2 mV (n = 28). As shown by the representative examples in Fig 7, after reaching a stable Vm recording in ECS, cells were sequentially exposed to ECS without 2 mM Ca^2+^ (nominally Ca^2+^ free ECS, arrow 1) for about 1 min, then 10 nM GSK101 in nominally Ca^2+^ free ECS (arrow 2) to activate TRPV4 without Ca^2+^ influx. Under these conditions, the activation of TRPV4 led to membrane depolarization because of Na^+^ influx through the activated TRPV4 channel. Upon the Vm depolarized to a new plateau, the perfusate was rapidly switched to the normal ECS with Ca^2+^ containing the same GSK101 concentration (10 nM GSK101, arrow 3). This allowed a rapid Ca^2+^ influx through the already activated TRPV4 channel, leading to a biphasic hyperpolarizing response: an initial very rapid instantaneous hyperpolarization that occurred within the first 2–3 sec (first hyperpolarization peak), and, for control cells, a slower secondary hyperpolarization occurring over several minutes. It is likely that the first phase represents rapid activation of KCa channels already residing at, or near, the plasma membrane while the second phase reflects either trafficking of additional KCa channels to the cell surface or possibly other secondary Ca^2+^-dependent changes. To determine the contributions of individual groups of KCa channels, selective KCa channel blockers were employed as follows: iberiotoxin (IbTX; 200 nM) for BKα [35, 36] (Fig 7B), TRAM-34 (300 nM) for IK1 [37] (Fig 7C), and Apamin (Apa; 300 nM) for SK1/SK3 [38, 39] (Fig 7D). The individual blockers were applied after the instantaneous hyperpolarization had fully developed as illustrated in Fig 7B–7E (arrows 4, 5, 6, 7, respectively).

We found that all KCa channel blockers tested, applied either individually or together (Fig 7E), typically resulted in an initial reduction in the first hyperpolarization peak, but then was followed by a slow suppression of the overall hyperpolarization over several minutes for all blockers (compare representative tracing in Fig 7B–7E to that for control cells in Fig 7A). Quantification of the hyperpolarization at 3 min following the blocker application (indicated by the arrowheads), normalized to the first hyperpolarization peak before the blocker, demonstrated significant inhibition by these blockers either applied individually or together (Fig 7F). In contrast to the additional 23.7 ± 9.0% increase in hyperpolarization at 3 min after Ca^2+^ re-addition to control cells (due to the second slow hyperpolarization phase), the hyperpolarization was reduced to 53.4 ± 11.6% by IbTX, 48.4 ± 11.7% by TRAM-34, 44.4 ± 9.7% by apamin, and 42.1 ± 10.4% by all 3 blockers applied together after the first hyperpolarization peak. Although it is not clear why the 3 blockers together did not exert a more potent effect than that for the individual blockers, the results strongly suggest that Ca^2+^ influx due to TRPV4 activation is functionally coupled to the stimulation of BKα, IK1, and SK1/SK3, consistent with immunocytochemistry demonstration for expression of each of these channels in the mCCDcl1 cells (Figs 3 and 4).

### Cross-Talk Between TRPV4 and KCa Channels

**Fig 8:**
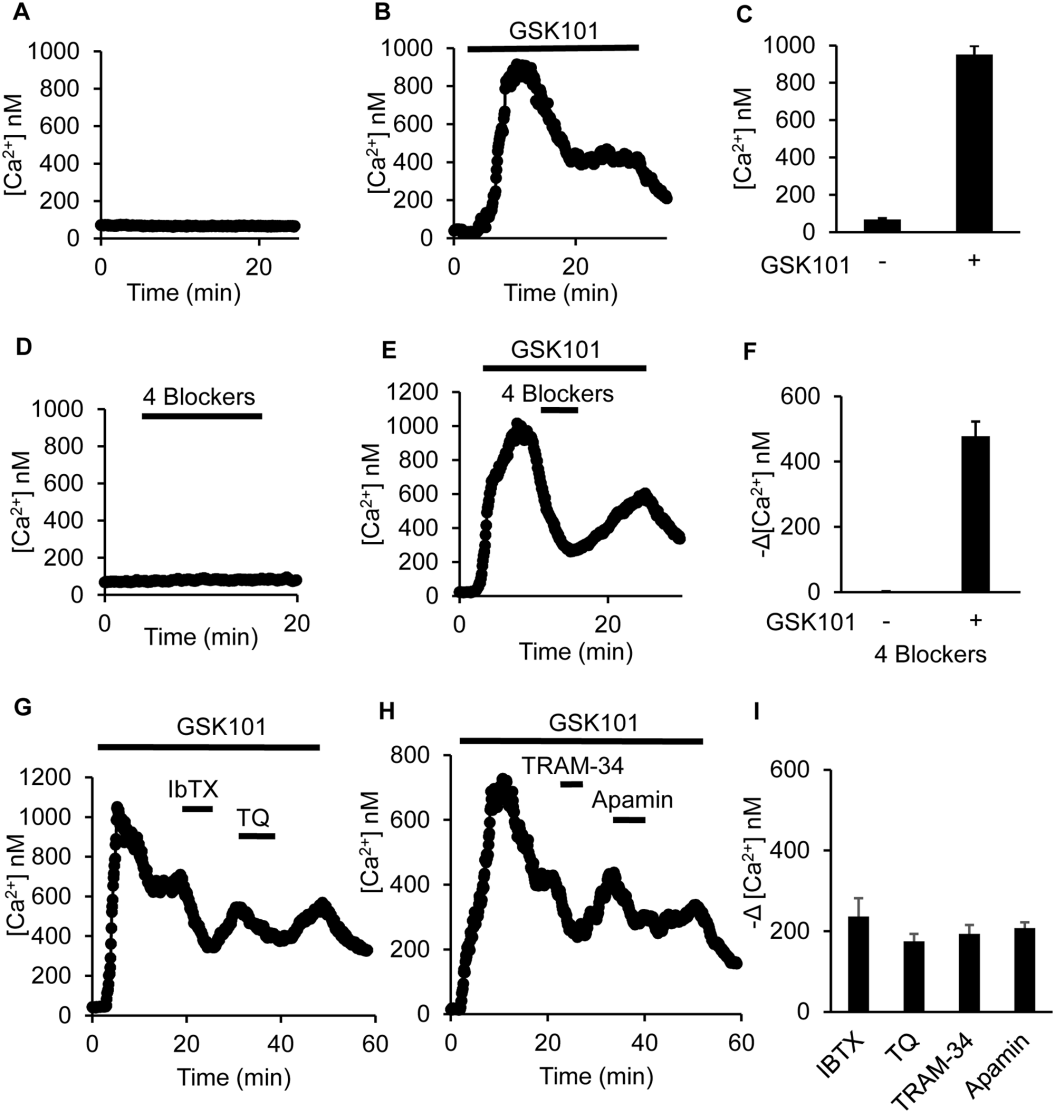
Measurement of intracellular Ca^2+^, [Ca^2+^]_i_, levels showing strong cross-talk between TRPV4 and the KCa channels in mCCDcl1. All traces are representative examples as indicated. Addition of GSK101 or the blockers is indicated by the length of the “bar” below each symbol in the individual tracings. **A.** In the absence of TRPV4 stimulation, [Ca^2+^]_i_ levels remain relative low in all cells. **B.** Upon activation of TPRV4 with the selective agonist, GSK101, [Ca^2+^]_i_ displays a biphasic response, rising to a peak [Ca^2+^]_i_ level with the first few minutes, then partial relaxes to a steady-state plateau which is above the basal [Ca^2+^]_i_ levels. **C.** The average changes in [Ca^2+^]_i_ are given showing the basal (GSK-) and peak (GSK+) [Ca^2+^]_i_ levels upon stimulation with GSK101 (3 nM). **D.** Effect of addition of a cocktail of KCa channel inhibitors (Apamin, 300 nM, IbTX, 100 nM, TRAM-34, 300 nM, and TQ, 5 nM) on [Ca^2+^]_i_ prior to activation of TRPV4 with GSK101. No response is apparent. **E.** Effect of addition of the same cocktail of KCa blockers as in panel **D**, but added after GSK101 activation of TRPV4. The cocktail of blockers markedly depressed the TRPV4-mediated [Ca^2+^]_i_ levels. **F.** Summary of the average decrease in [Ca^2+^]_i_ upon addition of the cocktail of KCa blockers (4 Blockers) before after activation of TRPV4. Addition of the 4 Blockers had not affect in the absence of GSK101 (-), averaging 0.4 ± 2.5 nM (n = 24). In the presence of GSK101 (+), the 4 Blockers induce a marked decrease in [Ca^2+^]_i_ of 477 ± 45 nM (n = 24, P<0.001) with the 4 Blockers, demonstrates a strong functional cross-talk between TRPV4, [Ca^2+^]_I_, and KCa channels. **G.** Effect of selective inhibition of BK (IbTX, 100 nM) or ROMK (TQ, 5 nM) on GSK101-induced [Ca^2+^]_i_ levels showing a significant reduction in [Ca^2+^]_i_ with either blocker. **H.** Effect of selective inhibition of IK1 (TRAM-34, 300 nM) or SKs (Apamin, 300 nM) on GSK101-induced [Ca^2+^]_i_ levels showing a significant reduction in [Ca^2+^]_i_ with either blocker. **I.** Summary results of the actions of each of the KCa blockers on GSK101-induced [Ca^2+^]_i_ levels showing a pronounced decrease in [Ca^2+^]_i_ levels with each of the blockers on TRPV4-mediated Ca2+ influx. The reduction in [Ca^2+^]_i_ averaged 236 ± 45 nM (n = 24) for IbTX, 175 ± 18 nM (n = 24) for TQ, 193 ± 22 nM (n = 24) for TRAM-34, and 208 ± 14 nM (n = 24) for Apamin (P<0.001 for each case). The individual responses do not differ among blockers. In contrast, the effect of the individual blockers is significantly less than that observed for the 4 Blocker combination in 8F (P<0.01).

The functional interplay between Ca^2+^-activated K^+^ channels and various Ca^2+^-permeable channels has long been known [51–53]. Since our patch clamp analysis demonstrated that Ca^2+^ influx mediated by TRPV4 can lead to activation of the KCa channels in mCCDcl1 cells, the hyperpolarization effect of the KCa channels may also facilitate Ca^2+^ influx mediated by TRPV4 because of the enhanced driving force. To test this, we monitored intracellular Ca^2+^ levels in mCCDcl1 cells grown on coverslips, using fura 2 quantitative fluorescence imaging of [Ca^2+^]_i_, as previously described [21, 27]. At low concentrations, from 3–10 nM, GSK101 rapidly induced Ca^2+^ influx as indicated by the rise in intracellular Ca^2+^ concentrations ([Ca^2+^]_i_) (Fig 8A and 8B). At 5 nM GSK101, the average [Ca^2+^]_i_ increased from a basal value of 67 ± 7 nM to a peak value of 952 ± 46 nM (Fig 8C). The response was similar to that previously shown for GSK101 induced activation of TRPV4 in CCD M-1 cells [27, 34] and split-opened CCD tubules [17, 23].

To assess the influence of K channels on TRPV4-mediated Ca^2+^ influx, we applied the KCa channel blockers as that used in the patch clamp experiments and in addition, tertiapin-Q (TQ; 5 nM), the blocker for the Ca^2+^-independent ROMK K^+^ channel [54–56], which represents the dominant resting K^+^ secretory channel in CNT and CCD. The inhibitors were added individually to the media or as a cocktail of all 4 blockers (4X Blockers).

In the absence of TRPV4 activation the addition of the K^+^ channel blockers had little effect on [Ca^2+^]_i_ values (Fig 8D). In contrast, after TRPV4 activation the blockers induced a dramatic decrease in [Ca^2+^]_i_ as shown by the representative example following addition of all 4 blockers (Fig 8E). The results are summarized in Fig 8F for addition of the 4 blockers on [Ca^2+^]_i_ prior to activation of TRPV4 (GSK101-) and after activation of TRPV4 (GSK101+). Prior to TRPV4 activation the effect of the blockers on [Ca^2+^]_i_ was negligible, whereas after TRPV4 activation the 4 blockers reduced [Ca^2+^]_i_ by 477 ± 45 nM. These results provide support for the concept of a strong cross-talk between TRPV4-mediated Ca^2+^ influx (GSK+) and activation of the K^+^ channels.

The effects of addition of single K^+^ channel blockers on [Ca^2+^]_i_ were less dramatic, but clearly apparent. This is evident from the representative examples shown for addition of IbTX or TQ alone (Fig 8G) and for TRAM-34 or Apamin alone (Fig 8H) after TRPV4 activation. The associated maximum decrease in [Ca^2+^]_i_ for each blocker is summarized for all experiments in Fig 8I with the decrease in [Ca^2+^]_i_ averaging 236 ± 45 nM for IbTX, 175 ± 18 nM for TQ, 193 ± 22 for TRAM-34, and 208 ± 14 nM for Apamin. However the effect of each of the blockers was significantly less than the 477 ± 45 nM change observed for all 4 Blockers combined (P<0.01, see Fig 8F). The studies clearly demonstrate a strong functional cross-talk between the TRPV4 channel, Ca^2+^ influx, and each of the KCa channels observed in the mCCDcl1 cells.

### Localization of Functional KCa Channels in CCD Cells

To further evaluate the functional location of TRPV4 and the K^+^ channels in CCD, we employed mCCDcl1 cells grown to confluency on permeable supports. With confluent monolayers on permeable supports, the transepithelial electrical resistance (TEER) can be measured and used to evaluate for activation or inhibition of ion channels if the channels are appropriately expressed at one or both cell borders (luminal versus basolateral borders). All measurements were performed in base media at 37°C (see Materials and Methods) after the monolayers (7–9 days) had reached a relatively high resting TEER value of at least 1000 Ω-cm^2^ which we use as an index of formation of a confluent monolayer with electrically “tight” tight junctions. At these high basal TEER values significant changes in ion channel activation (decreased TEER) and inhibition (increased TEER) at the cell membranes should be able to be observed.

**Fig 9:**
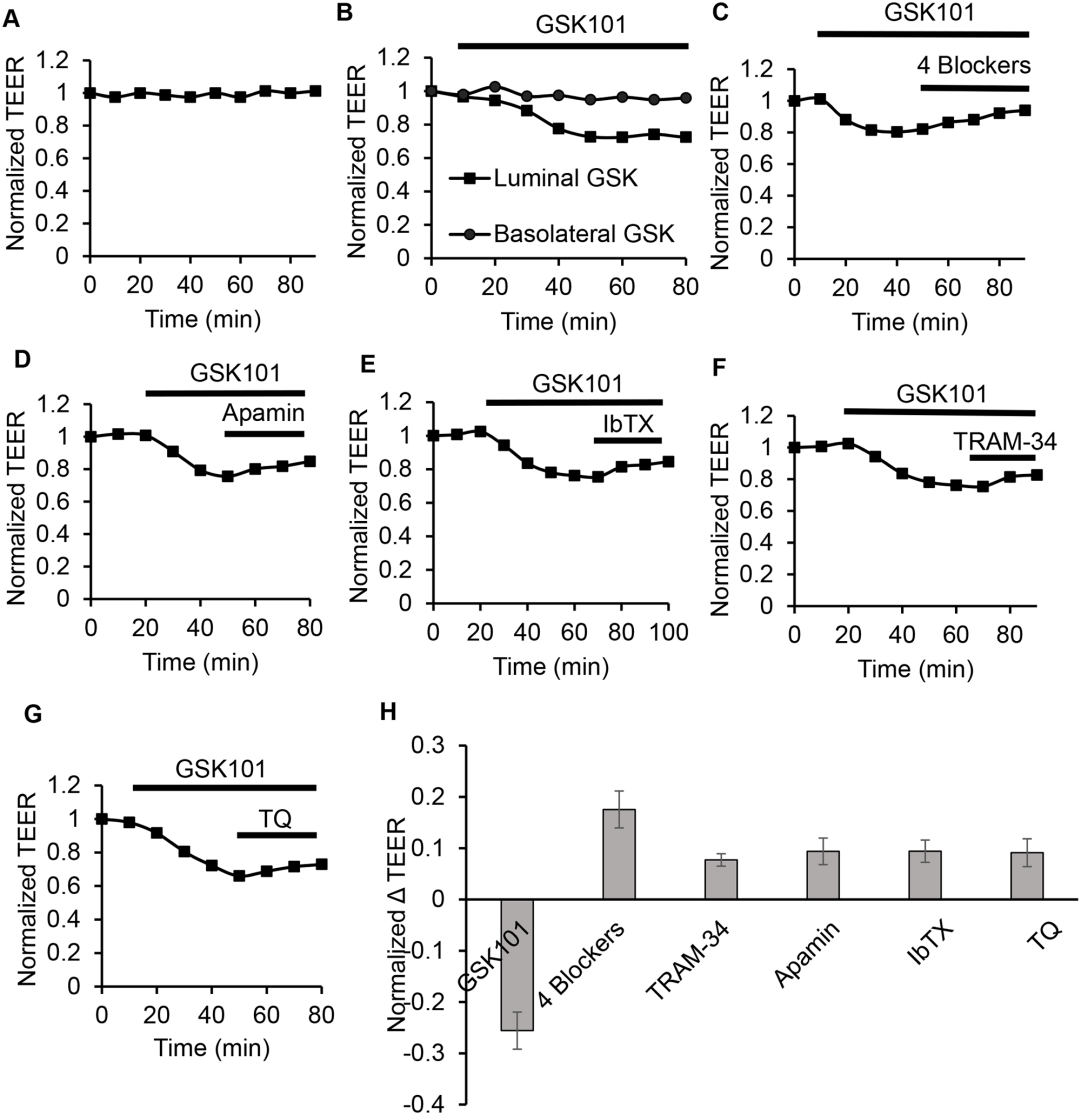
The effect of TRPV4 activation and KCa channel blockers on transepithelial electrical resistance (TEER) of mCCDcl1 cells grown to confluency on permeable supports (see Materials and Methods). Addition of GSK101 or the blockers is indicated by the length of the “bar” below each symbol in the individual tracings. **A.** Representative time course of TEER measurements under basal conditions showing stable TEER values over the time course of the experiments. **B.** Representative example of the effect of adding GSK101 to the outside basolateral compartment (Basolateral GSK) or to the luminal (apical) compartment (Luminal GSK). GSK101 had no apparent affect when added to the basolateral media, but showed a consistent decrease in TEER (reflecting activation of TRPV4 and other channels) when added to the luminal compartment. **C.** Example showing the effect of addition of the cocktail of KCa blockers (as in Fig 8) on the GSK101-induced reduction in TEER. Addition of the blockers lead to an increase in TEER levels, results consistent with inhibition of [Ca^2+^]_i_ activated KCa channels. **D.** Representative example showing the effect of SK inhibition (Apamin) on the GSK101-activated TEER values. **E.** Representative example showing the effect of BK inhibition (IbTX) on the GSK101-activated TEER values. **F.** Representative example showing the effect of IK1 inhibition (TRAM-34) on the GSK101-activated TEER values. **G.** Representative example showing the effect of ROMK inhibition (TQ) on the GSK101-activated TEER values. **H.** Summary results showing the effect of GSK101 activation of TRPV4 on TEER values (GSK, luminal compartment) and the subsequent individual KCa channel blockers (Luminal Blockers) on the TRPV4-activated TEER measurements. Each blocker (TRAM-34, Apamin, IbTX, TQ) or a cocktail (4X Blockers) of KCa channel blockers showed an increase in TEER. The decrease in TEER upon addition of GSK101 averaged 0.74 ± 0.027 (n = 3). The average increase in the relative TEER for the blockers in the presence of GSK101 was 0.17 ± 0.03 for the 4 Blockers, 0.08 ± 0.02 (n = 3) for TRAM-34, 0.09 ± 0.02 (n = 3) for Apamin, 0.09 ± 0.02 (n = 3) for IbTX, and 0.09 ± 0.02 (n = 3) TQ. Although the response to the 4 Blockers appears much larger, statistically there was no significant difference in the responses among the blocker sets.

High stable TEER values were achieved prior to each experiment and remained stable during the course of the experiments as shown by the representative example in Fig 9A. Indeed, basal TEER values averaged 3081 ± 151 Ω-cm^2^ under control conditions. Whereupon, all TEER values were normalized to the initial resting TEER value of 1 for all experiments. As shown by the representative example in Fig 9B, addition of the selective TRPV4 agonist, GSK101, to the basal side (outside) of the monolayers had little or no effect on TEER, likely indicating the TRPV4 channel function, if present, is at the luminal border. It should be noted that perfusion access to the basolateral compartment is very limited and, hence, any interpretation must be viewed with caution due to the limited solution exchange. In contrast, however, addition of GSK101 to the luminal side (inside, or apical side) of the monolayer was observed to induce a marked decrease in the relative TEER value from 1 to 0.74 ± 0.027 (n = 3, Fig 9B and 9H) pointing to functional TRPV4 channels at the luminal border. The luminal GSK101-induced decrease in the TEER values should reflect both activation of the TRPV4 channels and any functionally coupled Ca^2+^-activated K^+^ channels. Whereupon, we tested for the effect of each of the K^+^ channel selective blockers following TRPV4 activation. As noted, the K^+^ channel blockers were added to the luminal compartment only after the GSK101-induced decrease in TEER reach a stable low value as indicated by the example in Fig 9B. As shown for each of the representative experiments, addition of each of the K^+^ channel blockers induce an increase in TEER, consistent with inhibition of an active Ca^2+^-activated K^+^ channel (Fig 9D–9G). On average, the luminal addition of the blockers led to a significant increase in relative TEER of 0.17 ± 0.03 for 4 Blockers, 0.08 ± 0.02 for TRAM-34, 0.09 ± 0.02 for Apamin, 0.09 ± 0.02 for IbTX, and 0.09 ± 0.02 for TQ. Addition of all four blockers (4X Blockers) to the luminal compartment trended to a larger response, but was not a statistically significant difference over that for individual blockers under the conditions of these studies. These studies point to at least partial function of TRPV4, ROMK, and the KCa channels at the luminal membrane of mCCDcl1 cells, results consistent with that expected in CCD based on our immunolocalization studies (Figs 3, 5 and 6).

## Discussion

The expression and function of KCa channels along the nephron and collecting ducts are currently not well described. It has been shown, however, that the Ca^2+^-activated maxi-K^+^ channel (BK) is expressed in the collecting duct system (CDS) where it is known to play a key role in controlling K^+^ secretion in the CNT and CCD [9–11, 13–16, 18]. While ROMK underlies the basal rates of K^+^ secretion in these segments, BK appears to come into play during altered physiology/pathophysiological states, such as during enhanced tubular flow to the distal nephron, where Ca^2+^-dependent activation of BK leads to enhance K^+^ excretion and, typically, to K^+^ wasting (see Introduction). The current study demonstrates for the first time that, in addition to BK, an array of KCa channels are expressed in the CNT and CCD and that these channels, along with TRPV4, may play a central role in distal tubule cell function. Specifically, it was discovered that, along with BK, two small-conductance Ca^2+^-activated K^+^ channels, notably SK1 and SK3, and the intermediate conductance Ca^2+^-activated K^+^ channel, IK1 (the Gardos channel, [57]), were expressed in the CNT and CCD (Figs 2, 5 and 6). Further, our patch clamp analysis and TEER experiments demonstrate that these KCa channels can be rapidly activated upon induction of Ca^2+^ influx via the mechanosensitive TRPV4 channel as shown in the mCCDcl1 cells (Figs 7, 8 and 9). In a prior study we had noted that SK3 was expressed in these segments of the mouse kidney and was functionally regulated by TRPV4 activation [23]. Hence, there appears to be an array of KCa channels that are expressed in the CNT and CCD and regulated by TRPV4-mediated Ca^2+^ influx.

The functions of this newly discovered array of KCa channels in the renal collecting duct system is not fully understood at this point, but the observed interplay amongst channels would point to a central control of K^+^ balance through regulation of K^+^ secretion. Indeed, It has been shown in other tissues that such arrays of KCa ion channels can lead to functional “collaboration” among Ca^2+^ channels and the various KCa channels (see reviews [51, 53, 58–61]). Indeed, diverse arrays of BK, SK, and IK channels have recently been shown to be linked to both voltage- and non-voltage-activated Ca^2+^ channels in a wide range of tissues and cell types. These range from central nervous system neurons and control of the after hyperpolarization potential [62–64], to vascular endothelial/smooth muscle cells and control of blood pressure and endothelial injury [65–67], to exocrine glands and control of fluid secretion [68, 69], to name a few. Indeed, in vascular endothelial cells it has been shown that flow/pressure-induced activation of TRPV4 in the vascular endothelial cells leads to activation of SK3 and IK1, and possible BK, which, in turn, results in membrane hyperpolarization as part of the endothelial-derived hyperpolarizing factor (EDHF) response [66, 67].

Could it be that a similar coordination amongst TRPV4 and the array of KCa channels observed in these other tissues is also at work in the collecting duct system? This appears to be the case as the KCa channel array we have uncovered in the collecting duct system would appear to provide a close functional collaboration between TRPV4 and the collecting duct KCa channels. Indeed, we have observed a tight cross-talk between TRPV4-mediated Ca^2+^ entry and activation of the various KCa channels (Figs 7 and 8). This cross-talk is particular apparent in the mCCDcl1 cells as the collaborative array of channels appear to be expressed in the same cells—as also appears to be the case for the cells of the CNT and CCD (Fig 6; see [23])—although we do see some differential expression of channels between PC and IC as noted (see below). While this functional arrangement may lead to regulation of various cell functions, we show that it specifically sets these channels up as modulators of the membrane potential, Vm, and Ca^2+^ influx that would, in turn, support enhanced K^+^ secretion via the luminal BK channel once activated by Ca^2+^ influx.

The high Ca^2+^-binding affinity of SK1/3 and IK1 channels, along with TRPV4, sets these channels up as “early” modulators of Vm and Ca^2+^ influx. Indeed, it has been shown that both the SK channels and IK1 channel constitutively bind calmodulin to a calmodulin binding domain (CaMBD) located in the C-terminus. Gating is controlled by Ca^2+^ binding to CaM which bestows upon this group of channels a very high Ca^2+^ binding affinity (EC_50_ = 300–600 nM, [70–72]). In contrast, for BKα the dominant Ca^2+^ binding site responsible for Ca^2+^-gating of the channel is a short stretch of aspartate residues in the C-terminus called the “Ca^2+^ bowl.” The site is characterized by a relatively low Ca^2+^ binding affinity (EC_50_ = 1–11 μM, [73–75]), an affinity in the range reported for BK in the CNT/CCD cells [9, 10]. However, BKα association with BKβ subunits can alter this affinity, being lowered by BKβ1 in particular, as noted for BKβ1 expression in the PC-like cell of the CNT [16, 76]. Nonetheless, with the much higher Ca^2+^ binding affinity, the SK1/3 and IK1 channels are anticipated to be activated early on during the initial activation of TRPV4, leading to an early hyperpolarization of Vm that would further enhance Ca^2+^ influx via TRPV4, as observed (Fig 8). This would occur regardless of whether these K^+^ channels were functional at the luminal or basolateral border of the cells. The enhanced Ca^2+^ influx associated with activation of SK and IK channels would serve to initiate or enhance activation of the low Ca^2+^-affinity luminal BKα channels leading to enhanced BKα-mediated K^+^ secretion. Further, as heretofore noted, the expression of the BKβ1 subunit is known to increase the Ca^2+^ sensitivity of the BK channel [77–79], as shown in a series of experiments by Sansom and coworkers [16, 76]. In these later studies BKβ1 appears to be predominantly, but not solely, expressed in the CNT, and could thereby readily also increased the sensitivity of the BK channel to Ca^2+^. This, again, would serve to contribute to an early activation of BK in this first CDS segment [76].

In view of the high Ca^2+^-binding affinity of the SK and IK channels, these channels may act more as “Ca^2+^ sensors” and/or modulators of [Ca^2+^]_i_. In contrast, the BK channel, with its lower Ca^2+^-binding affinity and strategic location at the luminal membrane, would function as the primary effector site for K^+^ secretion being regulated by TRPV4-mediated Ca^2+^ influx and the enhanced Ca^2+^ influx associated with early activation of the SK and IK channels. This model would be consistent with enhance rates of CNT/CCD K^+^ secretion and K^+^ wasting during states of high distal tubule flow rates where both TRPV4 and BKα are already established as key players in this process [14–19]. It remains for future studies to explicitly define the extent to which the SK and IK channels serve as modulators of BK channels in this scenario and whether other factors, such as expression of channels in various microdomains or compartments, may play a role in this process.

Could the SK1/3 and IK1 channels also directly contribute to K^+^ secretion during states of elevated, Ca^2+^-dependent, K^+^ excretion? Clearly these channels may play a role beyond their sensory control of Vm and Ca^2+^ entry if they are expressed and functional at the luminal membrane of CDS cells. Our TEER measurements in mCCDcl1 cells grown on permeable supports provide initial evidence that these channels, or a fraction of these channels, are functional at the luminal border since luminal addition of these KCa channel blockers in our patch clamp studies and TEER analysis lead to a decrease in TRPV4-mediated Vm hyperpolarization and an increase in TEER (Figs 7 and 9). Further, our past immunostaining studies of SK3 in mouse kidney slices point to expression of SK3 at both the basolateral and luminal cell membranes of CNT and CCD [23]. Indeed, our current kidney immunohistochemical staining patterns observed for the array of K^+^ channels in CNT and CCD are consistent with this view (Fig 6), but do not speak directly to the functional site of expression. However, any SK1/3 and IK1 channel functionally expressed at the luminal border would, by design, contribute to net K^+^ efflux across the luminal border once the channel is activated. Recent studies of flow-induced K^+^ secretion in BKα and BKβ1 mouse knockout models clearly point to a dominant role of the BK channel in these K^+^ wasting states, although contribution from other K^+^ secretory channels seems likely [14–16]. To wit, it was observed in these earlier studies that under states of high flow the enhance K^+^ secretion is reduced to that anticipated for the basal ROMK-mediated K^+^ secretion. However, as noted in the Introduction, the high tubular flow is known to stimulate release of ATP into the lumen of the distal tubule [24, 25] which can inhibit the ROMK channel [26, 43] and, as a result, could lead to a reduced ROMK-mediated K^+^ secretion. Hence, participation of other flow/Ca^2+^-activated K^+^ channels in the K^+^ secretory processes seems likely even during states of BK gene ablation. The magnitude of this contribution, however, is currently not known.

Important to the general scheme for TRPV4 mediated control of the KCa channels in the CDS is, of course, the specific cell types that these channels are expressed in along the CNT and CCD. While we show in this study (Figs 5 and 6) and our previous study [17, 23], TRPV4 is expressed in all cell types of both the CNT (PC-like cells and IC) and CCD (PC and IC). However, the apparent expression of TRPV4 was higher in PC over IC, results consistent with that of the current study which we demonstrated previously leads to an enhanced TRPV4-mediated Ca^2+^ influx in PC over IC [17]. However, the expression of some of the KCa channels also shows differential expression levels. SK1 and SK3 are expressed in both CNT and CCD segments, but we show that SK1 expression dominants in IC while SK3 expression dominants in PC. In contrast, IK1 is also expressed in both CNT and CCD, but we show in CCD that it appears to be expressed in both PC and IC with a relative more dominant expression in PC (Fig 6). What this means to K^+^ secretion in CNT and CCD is not fully appreciated at this moment as this will require an more in depth assessment of SK1/SK3 and IK1 function in CDS.

In a similar manner we have shown in our studies that the main KCa channel, BKα, is strongly expressed in both CNT and CCD, and that based on CCD staining patterns it appears to be expressed to similar levels in both PC and IC as shown by our immunofluorescence studies. However, it has been reported by other laboratories that immunhistochemical staining for BKα and its associated BKβ1 subunit in mouse CDS, showed predominant staining in the CNT with more prominent staining of the PC-like connecting tubule cell over the IC [16]. While other similar studies demonstrated BKα expression in CCD, staining for BKα displayed preferentially high levels of staining in IC over PC in many studies [47–49]. This differs from our own study where immunostaining for BKα with two different primary antibodies showed BKα expression of both the CCD PC and IC. However, we did observe that one of the antibodies employed showed staining in both PC and IC, but with preferential staining of IC (Alomone APC107), while the second antibody (Alomone APC151) also display staining of both PC and IC, but with preferential staining of PC (see Figs 5D and 6D). Such differential labeling of the same channel subunit by two different primary antibodies is not completely surprising as, for example, the two Alomone anti-BKα antibodies that we employed were developed against two different epitopes (one extracellular, one intracellular). Differences in antigenic binding sites on each antibody (paratopes) would also differ as could the assembly/folding of the BKα into signaling plexes in PC versus IC, all leading to potential differential staining for one antibody over the other. Hence, depending on the primary antibody employed in the staining studies, preferential staining may account for some of the noted differences in the reported BKα expression levels in PC over IC.

Patch clamp analysis of functional BK activity in CNT and CCD also points to expression of functional BKα channels in both PC and IC cells. Indeed, while early patch clamp analysis of rat and mouse CNT and CCD reported BK activity on the luminal membrane of both IC and PC, although the BK channel activity was observed most frequently in IC [11, 12, 80]. These early observations clearly implicated the IC as the dominant cell type with functionally BK channels. However, it was subsequently shown that while BK channel activity was low in PC under basal conditions, the BK channel activity in PC could readily be upregulated to that observed in IC by either inhibition of the MAPK pathway [81] or upon activation of the cytochrome P450 expoxygenase pathway via application of arachidonic acid (AA) or its downstream epoxyeicosatrienoic acid metabolite, 11,12-EET [82]. Indeed, since the cytochrome 450 expoxygenase pathway has been shown to, in part, underlie mechanical/flow-induced activation of TRPV4 [83, 84] and KCa channels of vascular endothelial cells [66, 85–87], this regulated pathway could be a central component in regulating flow-induced K^+^ secretion in CNT and CCD. Hence, it is apparent that BK is functionally expressed in both PC and IC of the CNT and CCD and that regulation of this channel in PC may be an important component in flow-dependent K^+^ secretion in the CCD. Our understanding of how TRPV4 and the other KCa channels may contribute to this regulation in IC versus PC remains under active investigation.

Finally, it is also evident from our studies that the mCCDcl1 cell line may be a potential strong cell model for evaluating the interplay between TRPV4 and the identified Ca^2+^-activated K^+^ channels in renal cells. This cell line was originally demonstrated to secrete K^+^, unlike other cell lines (e.g., M-1 cells, [43]; mpkCCD cells, [30]), and express ROMK, setting this cell line up as a potential model for K^+^ transport of the kidney CNT and CCD [30, 31]. We have now shown that these cells not only express functional TRPV4 channels at the luminal border, but they express the same array of KCa channels that we demonstrate are expressed in the mouse CNT and CCD (Figs 1–6). In addition, the mCCDcl1 cells have been shown to be excellent models of Na^+^ reabsorption mediated by ENaC [31, 41]. Hence, the cells would appear to support many of the electrolyte transport properties of CCD PC which will make these cells particularly amenable for assessing many aspects of electrolyte transport in the late distal tubule.

In summary, the current studies demonstrates that mCCDcl1 cells and cells of the CNT/CCD express both TRPV4 and an array of KCa channels including small conductance (SK1, SK3), intermediated conductance (IK1), and large conductance (BK) Ca^2+^-activated K^+^ channels. The array of channels display a high level of cross-talk between TRPV4 and all of the KCa channels, pointing to collaborative control of Ca^2+^ influx, membrane potential, and K^+^ secretion. This collaborative arrangement of ion channels expressed in the collecting duct system will bring about tight control of [Ca^2+^]_i_ and flow-dependent K^+^ secretory rates in the distal tubule and, hence, may play a critical role in generating K^+^ wasting states associated with inappropriate states of K^+^ imbalance.
